# Increased NFATC4 Correlates With Poor Prognosis of AML Through Recruiting Regulatory T Cells

**DOI:** 10.3389/fgene.2020.573124

**Published:** 2020-11-27

**Authors:** Chong Zhao, Shaoxin Yang, Wei Lu, Jiali Liu, Yanyu Wei, Hezhou Guo, Yanjie Zhang, Jun Shi

**Affiliations:** ^1^Department of Hematology, Shanghai Jiao Tong University Affiliated Sixth People's Hospital, Shanghai, China; ^2^Department of Hematology, Shanghai Ninth People's Hospital, Shanghai Jiao Tong University School of Medicine, Shanghai, China

**Keywords:** acute myeloid leukemia, NFATc4, regulatory T cells, immune response, prognosis

## Abstract

Despite that immune responses play important roles in acute myeloid leukemia (AML), immunotherapy is still not widely used in AML due to lack of an ideal target. Therefore, we identified key immune genes and cellular components in AML by an integrated bioinformatics analysis, trying to find potential targets for AML. Eighty-six differentially expressed immune genes (DEIGs) were identified from 751 differentially expressed genes (DEGs) between AML patients with fair prognosis and poor prognosis from the TCGA database. Among them, nine prognostic immune genes, including NCR2, NPDC1, KIR2DL4, KLC3, TWIST1, SNORD3B-1, NFATC4, XCR1, and LEFTY1, were identified by univariate Cox regression analysis. A multivariable prediction model was established based on prognostic immune genes. Kaplan–Meier survival curve analysis indicated that patients in the high-risk group had a shorter survival rate and higher mortality than those in the low-risk group (*P* < 0.001), indicating good effectiveness of the model. Furthermore, nuclear factors of activated T cells-4 (NFATC4) was recognized as the key immune gene identified by co-expression of differentially expressed transcription factors (DETFs) and prognostic immune genes. ATP-binding cassette transporters (ABC transporters) were the downstream KEGG pathway of NFATC4, identified by gene set variation analysis (GSVA) and gene set enrichment analysis (GSEA). To explore the immune responses NFATC4 was involved in, an immune gene set of T cell co-stimulation was identified by single-cell GSEA (ssGSEA) and Pearson correlation analysis, positively associated with NFATC4 in AML (*R* = 0.323, *P* < 0.001, positive). In order to find out the immune cell types affected by NFATC4, the CIBERSORT algorithm and Pearson correlation analysis were applied, and it was revealed that regulatory T cells (Tregs) have the highest correlation with NFATC4 (*R* = 0.526, *P* < 0.001, positive) in AML from 22 subsets of tumor-infiltrating immune cells. The results of this study were supported by multi-omics database validation. In all, our study indicated that NFATC4 was the key immune gene in AML poor prognosis through recruiting Tregs, suggesting that NFATC4 might serve as a new therapy target for AML.

## Introduction

Acute myeloid leukemia (AML) is the most common type of acute leukemia in adults, which often confronts high recurrence risk and low 5-years survival after diagnosis (Li et al., 2020). Over the past decades, therapies targeting mutated or critical proteins in leukemia have come to the market with some promising impact on prognosis (Pollyea, 2018; Cerrano and Itzykson, 2019). However, immune therapy which has gained significant clinical impact on other neoplastic diseases still faces great challenges in AML. This indicates us to pay more attention to immune regulation in AML.

The progression of AML is closely associated with immune imbalance. As important participants in immune responses, changes in the type and proportion of immune cells are involved in cancer progression. The percentage of regulatory T cells (Tregs) in bone marrow is higher in AML patients than in healthy donors (Niedzwiecki et al., 2019; Williams et al., 2019). Increased Treg phenotype may promote disease progression and lead to poor prognosis in AML through contributing to immune evasion (Govindaraj et al., 2014; Arandi et al., 2018). However, how immune cells involved in immune imbalance are regulated in AML remains unclear.

Immune genes in tumor cells may promote the secretion of inflammatory cytokines by activating the downstream signaling pathway and recruit Tregs, thus avoiding immune damage (Yue et al., 2020). Therefore, we screened immune genes from ImmPort to study how immune genes in leukemia cells regulate immune responses in AML. Limiting the target to immune genes might help us identify immune factors associated with AML prognosis more accurately. In this study, we identified key immune genes correlated with AML prognosis and explored the associated immune gene set and immune cells by ssGSEA and CIBERSORT algorithm with the expression profiles from the TCGA database, trying to find novel targets for immunotherapy.

## Materials and Methods

### Data Preparation and Analysis of Differentially Expressed Genes (DEGs)

RNA-seq profiles and clinical information of AML samples with different risk stratifications were downloaded from The Cancer Genome Atlas (TCGA) database (https://tcgadata.nci.nih.gov/tcga/). Primary AML samples with complete clinical information and not M3 subtype were selected for our following analysis. Data of 2,498 immune-related genes were retrieved from the ImmPort database (https://www.import.org/) (Bhattacharya et al., 2018). Data of 318 cancer-related transcription factors (TFs) were obtained from the Cistrome Cancer database (http://cistrome.org/) (Mei et al., 2017). HTseq-count and fragments per kilobase of exon per million reads mapped (FPKM) profiles of AML samples, divided into two groups with fair prognosis (risk stratification: favorable/intermediate) and poor prognosis (risk stratification: poor), were assembled. To identify significantly DEGs, the edgeR method was used (Robinson et al., 2010) while *P* < 0.05 and the log (fold change) > 1 or < −1 were set as the cutoffs. The heatmap showed the DEGs with each row normalized by z-score. The volcano plot was generated to highlight DEGs. Gene Ontology (GO) and Kyoto Encyclopedia of Genes and Genomes (KEGG) enrichment analysis of DEGs were performed to reveal the potential mechanism.

### The Identification of Prognostic Immune Genes

DEIGs were extracted from the previously identified DEG list and immune-related genes. Heatmap and volcano plot were applied to show the DEIGs. Then, the univariate Cox regression analysis was performed to identify prognostic immune genes based on DEIGs and clinical information.

### Construction of Prognostic Prediction Model Based on the Prognostic Immune Genes

To assess the significance of each prognostic immune gene with the β-value, the multivariate Cox regression analysis was carried out. Based on the model, the risk score of each sample was calculated to evaluate prognostic risk according to the following formula:

$$\begin{array}{l} \text{Risk score}=\overset{n}{\underset{i=1}{\sum }}\text{βi}\times xi \end{array}$$

In the formula, “*n*” represents the number of integrated genes in the model. “β” represents the regression coefficient of each integrated gene. “χ” represents the expression level of each integrated gene. Then, based on the median risk score, samples were medially divided into high- and low-risk groups. The area under the ROC curve (AUC) was applied to evaluate the accuracy of the model. Kaplan–Meier survival analysis was performed to compare the survival between the two groups. Next, based on the risk score, individuals were reordered. The risk curve, survival state-related scatterplot, and heatmap of prognostic immune genes were plotted.

Then, to assess the independent prognostic value of the risk score, age, gender, morphology code (FAB subtype), and risk category, the univariate and multivariate Cox regression analyses modified by baseline information were performed.

### The Identification of the Key Immune Gene

Differentially expressed transcription factors (DETFs) were obtained by intersecting DEGs and all the cancer-related TFs, shown by the heatmap and volcano plot. Then, Pearson correlation analysis was conducted to uncover the regulation and association between DETFs and prognostic immune genes. The regulation pair with the highest coefficient and *P* < 0.05 was selected as the significant regulation pair. The immune gene in the significant regulation was recognized as the key immune gene. The expression of the key immune gene in different AML risk stratifications was shown in the box plot.

### Identification of Potential Downstream KEGG Pathways, Immune Gene Sets, and Immune Cells

To explore the downstream KEGG pathways of key immune gene related to AML prognosis, gene set variation analysis (GSVA) was performed. ssGSEA was applied to identify the immune gene sets related to AML prognosis from those overexpressed in the tumor microenvironment (Barbie et al., 2009; Charoentong et al., 2017). CIBERSORT was used to quantify the proportions of immune cells related to AML prognosis (Newman et al., 2015). Pearson correlation analysis was performed to clarify the correlation relationship between key immune gene and KEGG pathways, immune gene sets, and immune cells, shown by the co-expression heatmap. The correlation scores were fitted by linear regression. Meanwhile, GSEA was also performed to find out the critical KEGG pathway. The overlap of GSVA and GSEA was shown by the Venn plot. The KEGG pathways identified by both GSVA and GSEA were recognized as the key signaling pathway.

### Multidimensional Validation and Construction of the Protein–Protein Interaction Network

With the aim of decreasing the bias based on different platforms, multidimensional validation was utilized. Moreover, genes that represented the KEGG pathway were available from Pathway Card (https://pathcards.genecards.org/). The databases of Gene Expression Profiling Interactive Analysis (GEPIA) (Tang et al., 2017), Oncomine (Rhodes et al., 2004), PROGgeneV2 (Goswami and Nakshatri, 2014), UALCAN (Chandrashekar et al., 2017), Linkedomics (Vasaikar et al., 2018), cBioportal (Cerami et al., 2012), Genotype-Tissue Expression (GTEx) (Consortium, 2015), UCSC xena (Goldman et al., 2015), Cancer Cell Line Encyclopedia (CCLE) (Ghandi et al., 2019), Expression atlas (Papatheodorou et al., 2018), The Human Protein Altas (Uhlen et al., 2015), and String (Snel et al., 2000) were applied to validate the scientific hypothesis.

To better reveal the mechanism related to AML prognostic status, a protein–protein interaction (PPI) network was built to illustrate the interaction among prognostic TF, immune genes, KEGG pathways, immune gene sets, and immune cells by Cytoscape 3.7.1 (Shannon et al., 2003). Accordingly, a signaling diagram was displayed to show the AML prognostic related hypothesis based on the bioinformatics.

### Statistical Analysis

R version 3.5.1 (Institute for Statistics and Mathematics, Vienna, Austria; https://www.r-project.org) was used for all the statistical analyses. For descriptive statistics, mean ± standard deviation (*SD*) was used to express the continuous variables in normal distribution while the median (range) was used in abnormal distribution. Classified variables were expressed by counts and percentages. Only two-tailed *P* < 0.05 was considered statistically significant.

## Results

### Nine Prognostic Immune Genes Were Identified in AML

**Figure 1:**
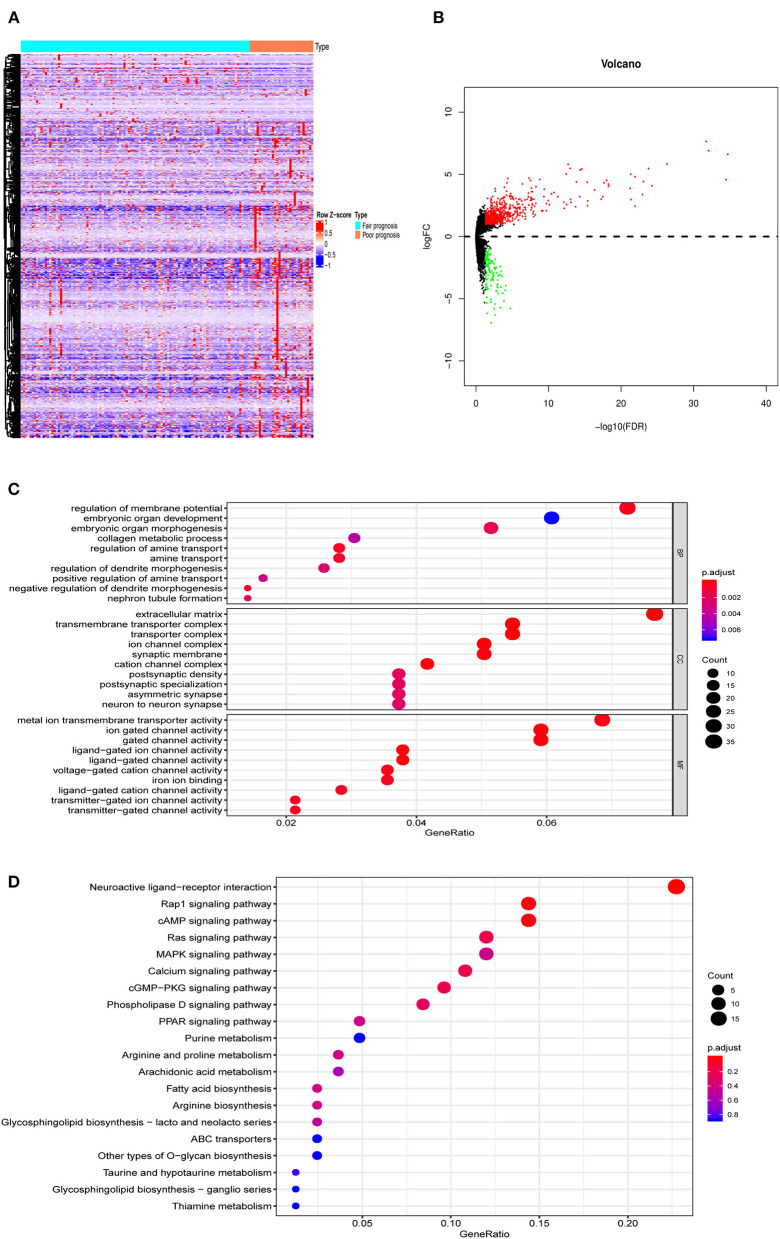
The DEGs between AML with fair and poor prognosis. **(A)** The heatmap and **(B)** volcano plot of 751 DEGs between 105 AML with fair prognosis and 29 AML with poor prognosis. **(C)** The GO and **(D)** KEGG analysis of 751 DEGs. Note: Fair prognosis, risk stratification: favorable/intermediate; poor prognosis, risk stratification: poor. AML, acute myeloid leukemia; DEGs, differentially expressed genes; GO, Go Ontology; KEGG, Kyoto Encyclopedia of Genes and Genomes.

The analysis procedure is shown in Supplementary Figure 1. There were 134 AML patients meeting the inclusion criteria that consisted of 105 with fair prognosis and 29 with poor prognosis. The baseline information is presented in Table 1. DEGs between the two groups, including 630 up- and 121 down- genes, were shown by the heatmap and volcano plot (Figures 1A,B). Then, GO and KEGG analyses were performed to reveal the underlying mechanism. As shown in Figures 1C,D, immune-related pathways such as “MAPK signaling pathway” and “ABC transporters” were included in the top 10 enrichment items.

**Table 1:** Baseline information of 134 patients with AML from the TCGA database.

**Variables**	**Total patients (*N* = 134)**
**Age, Years**	
Mean ±*SD*	55 ± 33
**Gender**	
Female	60 (44.8%)
Male	74 (55.2%)
**Risk Stratification**	
Favorable/intermediate	105 (78.4%)
Poor	29 (21.6%)
**Morphology Code**	
M0	15 (11.2%)
M1	34 (25.4%)
M2	38 (28.3%)
M4	28 (20.9%)
M5	15 (11.2%)
M6	2 (1.5%)
Other	2 (1.5%)

*SD, Standard deviation*.

**Figure 2:**
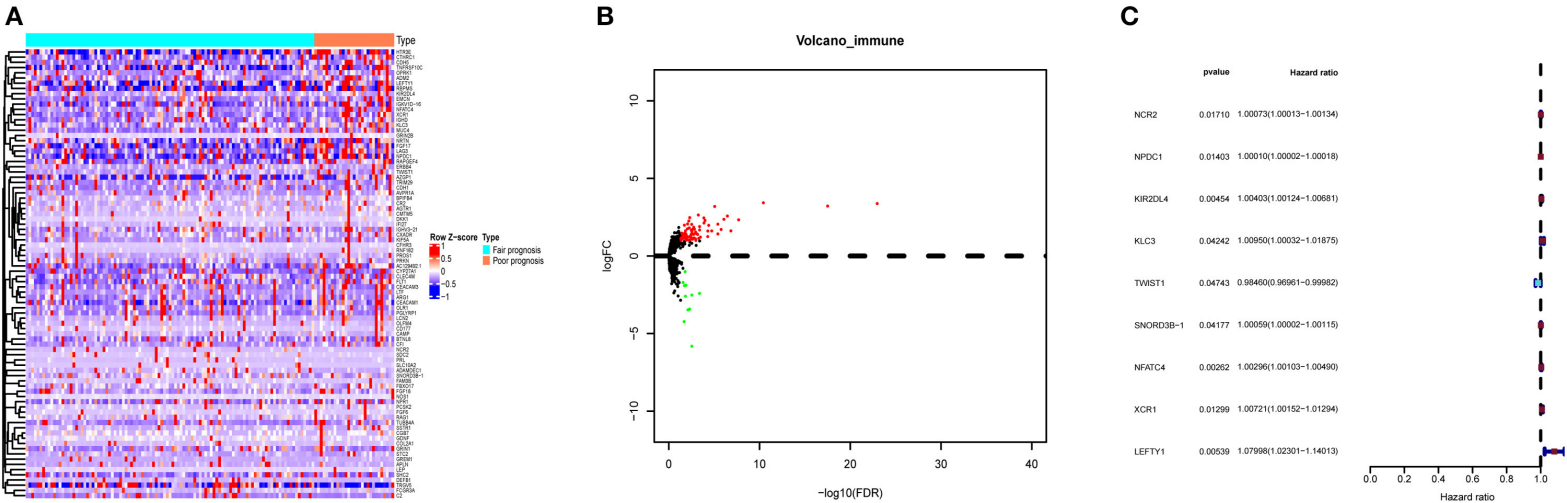
The DEIGs between AML with fair and poor prognosis. **(A)** The heatmap and **(B)** volcano plot of 86 DEIGs; **(C)** forest plot to show the nine prognostic immune genes. Red: high-risk gene; blue: low-risk gene. DEIGs, differentially expressed immune genes.

DEIGs (75 up- and 11 down- genes) were shown by the heatmap and volcano plot in Figures 2A,B. To find out prognostic immune genes, the DEIGs and prognosis data were sent for univariate Cox regression analysis. Nine prognostic immune genes, including NCR2, NPDC1, KIR2DL4, KLC3, TWIST1, SNORD3B-1, NFATC4, XCR1, and LEFTY1, were identified (Figure 2C).

To make our results more convincing, we also divided AML patients in three groups including favorable, intermediate, and poor prognosis for nonparametric tests. As shown in Supplementary Figure 2, most of the prognostic immune genes did differ among the three groups (*P* < 0.05).

**Figure 3:**
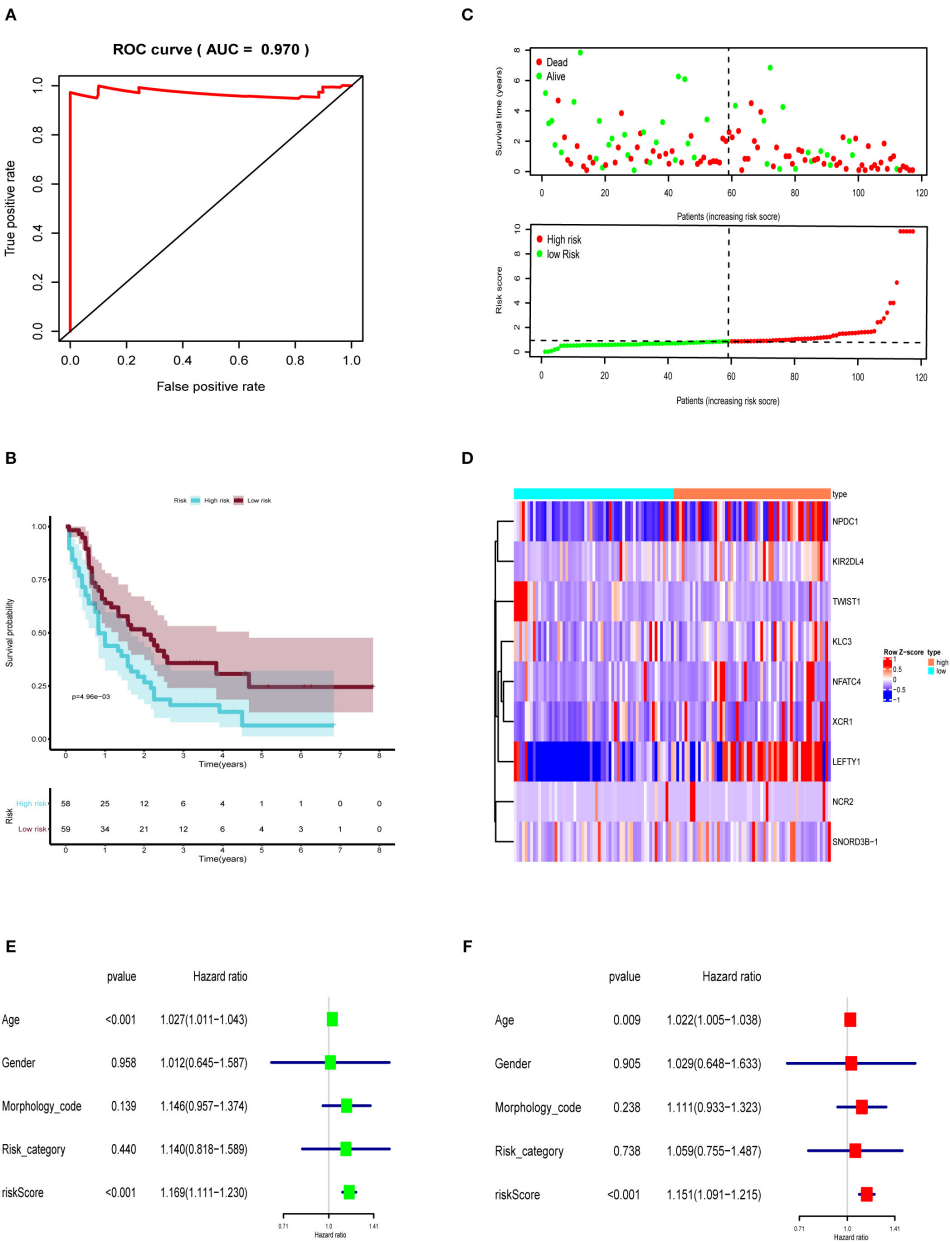
The prognostic prediction model based on prognostic immune genes. **(A)** ROC curve for (AUC = 0.970) prognostic immune genes. **(B)** The Kaplan–Meier curve to identify the efficacy of risk score in OS. **(C)** The high and low risk score group in scatterplot and risk plot. **(D)** The heatmap to illustrate each prognostic immune genes screened by Lasso regression. The forest plot of univariate **(E)** and multivariate **(F)** Cox regression analysis. OS, overall survival; AUC, area under the curve.

Then, the prognostic immune genes and clinical information were integrated into a multivariate Cox regression analysis to establish the prognostic prediction model. The Lasso regression was performed to avoid overfitting of the model. The AUC was 0.970 in the ROC curve, indicating that all these nine genes were essential for modeling (Figure 3A). The risk score of each sample was calculated accordingly. Individuals were divided into two groups with high and low risk with the median value of 1.000. The Kaplan–Meier curve showed that the survival probability of samples in the high-risk group was significantly lower than in the low-risk group (*P* < 0.001), suggesting good effectiveness of the prediction model (Figure 3B).

Then, the risk curve and scatterplot were generated to show the risk score and survival status of each individual with AML. Patients in the high-risk group showed higher mortality than those in the low-risk group, as shown in Figure 3C. The expression of prognostic immune genes screened by Lasso regression were displayed by the heatmap in Figure 3D.

To assess the independent prognostic value of risk score, we sent age, gender, morphology code, risk category, and risk score to the univariate and multivariate Cox regression analysis. As shown in Figures 3E,F, both the univariate (HR = 1.169, 95% CI (1.111–1.230), *P* < 0.001) and multivariate (HR = 1.151, 95% CI (1.091–1.215), *P* < 0.001) Cox regression analyses indicated that the risk score was an independent prognostic factor in AML.

### NFATC4 Was the Key Immune Gene Associated With Poor Prognosis of AML

**Figure 4:**
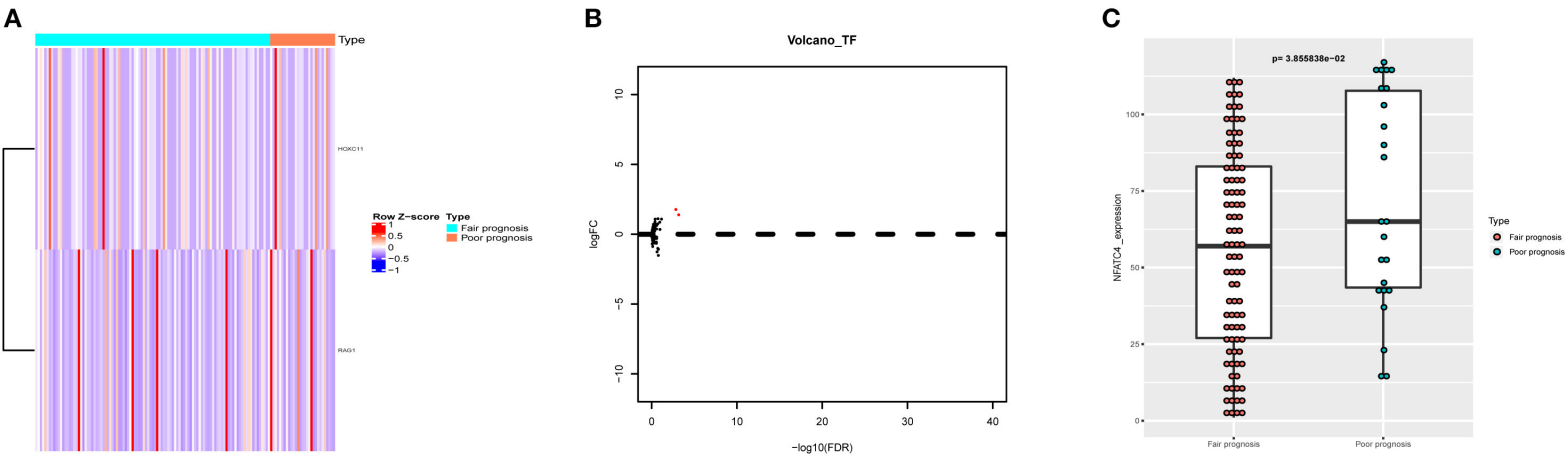
The DETFs between AML with fair prognosis and poor prognosis. **(A)** The heatmap and **(B)** volcano plot of 2 DETFs. **(C)** The box plot to show the expression of NFATC4 in AML with different prognostic statuses. DETFs, differentially expressed transcription factors; RAG1, recombination activating gene-1; NFATC4, nuclear factor of activated T cells-4.

To further find out the critical immune gene related to poor prognosis of AML, the co-expression analysis of DETFs and prognostic immune genes was performed. Two up-DETFs between AML patients with fair prognosis and poor prognosis were displayed with the heatmap and volcano plot in Figures 4A,B. Then, Pearson correlation analysis between DETFs and prognostic immune genes was carried out. As shown in Table 2, only the pair of recombination activating gene-1 (RAG1) and nuclear factors of activated T cells-4 (NFATC4) was significant (*R* = 0.248, *P* < 0.01, positive), suggesting that RAG1 upregulated NFATC4 in AML. NFATC4 was recognized as the key immune gene. The expression of NFATC4 in different AML prognostic statuses is shown in Figure 4C. Patients with poor prognosis showed higher expression of NFATC4.

**Table 2:** The correlation analysis results of DETFs and prognostic immune genes.

**TF**	**Immune gene**	**Correlation**	***P*-value**	**Regulation**
RAG1	NFATC4	0.247618294	0.007108929	Positive

*TF, transcription factor; RAG1, recombination activating gene-1; NFATC4, nuclear factors of activated T cells-4*.

### NFATC4 Was Co-expressed With ATP-Binding Cassette (ABC) Transporter Signaling Pathway in AML Poor Prognosis

**Figure 5:**
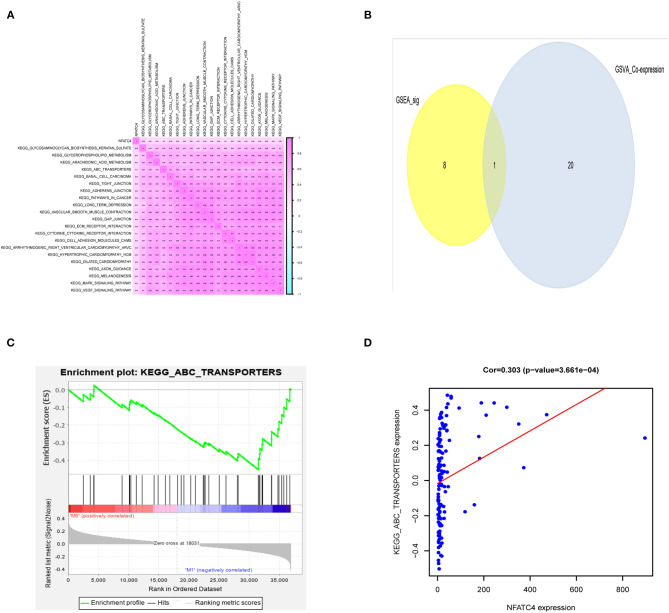
The downstream KEGG pathways of NFATC4 related to AML prognosis. **(A)** The co-expression heatmap of NFATC4 with KEGG pathways selected by GSVA. **(B)** The Venn plot to show overlap KEGG pathways in both GSVA and GSEA. **(C)** The GSEA analysis of ABC transporter pathway. **(D)** The linear regression to show the correlation between NFATC4 and ABC transporters pathway. GSVA, gene set variation analysis; GSEA, gene set enrichment analysis; ABC transporters, ATP-binding cassette transporters.

To explore the potential mechanism of NFATC4 regulating AML prognosis, GSVA was performed and a total of 21 KEGG signaling pathways related to AML poor prognosis were identified. Then, Pearson correlation analysis was carried out to construct the correlation relationship between NFATC4 and prognosis-related KEGG pathways (Figure 5A). Meanwhile, to identify the key KEGG pathway mostly correlated with AML prognosis, GSEA was also conducted. The pathways identified by GSVA and GSEA were intersected. The overlap in GSVA and GSEA was shown by the Venn plot. As shown in Figure 5B, there was only one pathway significant in both GSEA and GSVA. The GSEA analysis of the ABC transporter pathway is shown in Figure 5C (*P* < 0.001). The correlation relationship between the NFATC4 and ABC transporter pathway was displayed by linear regression in Figure 5D (*R* = 0.309, *P* < 0.001, positive), suggesting that NFATC4 might positively regulate ABC transporters in AML.

### NFATC4 Was Co-expressed With Immune Gene Set of T Cell Co-stimulation, Tregs in AML

**Figure 6:**
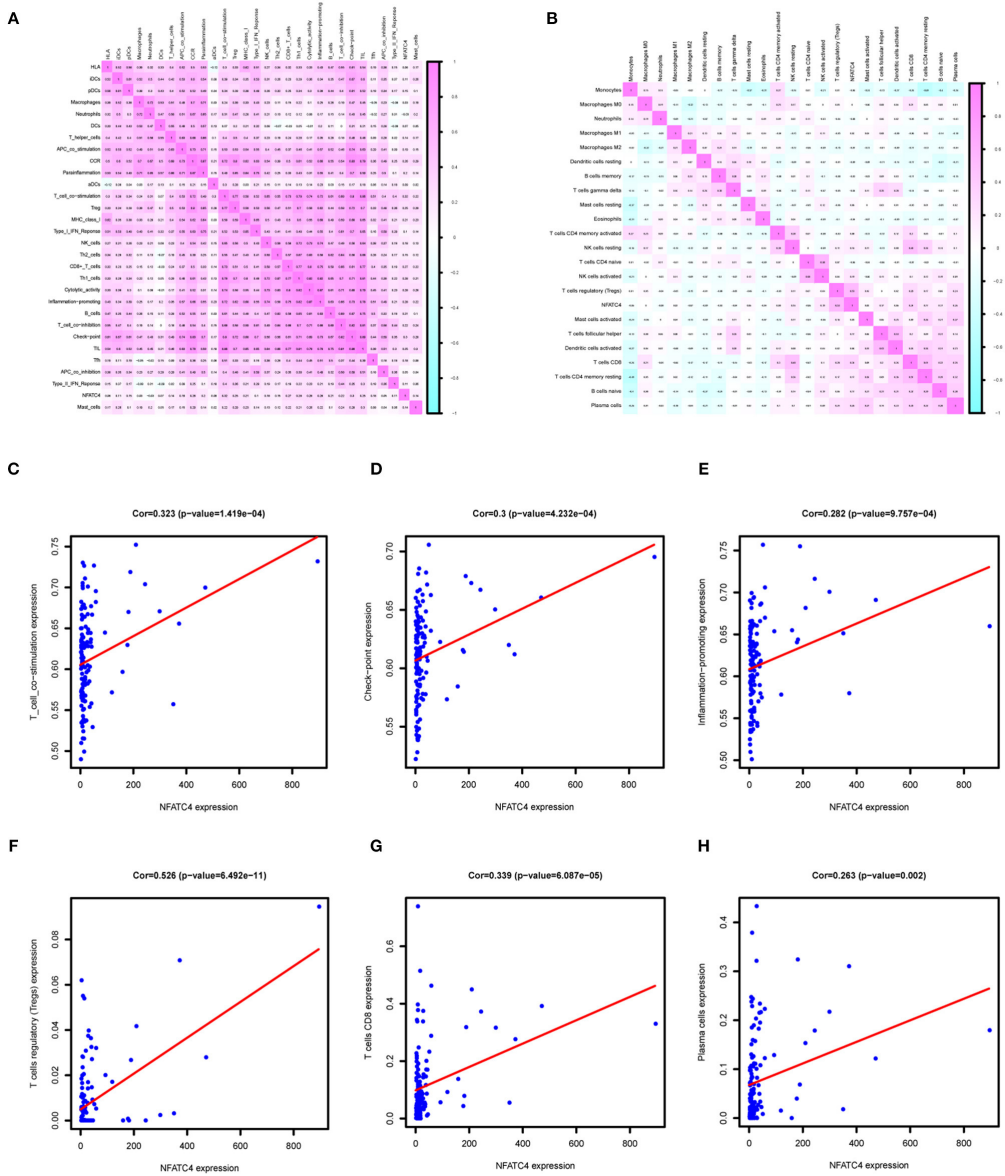
The immune gene sets and immune cells related to NFATC4 in AML. The co-expression heatmap of NFATC4 with **(A)** immune gene sets and **(B)** immune cells. The linear regression to show the correlation between NFATC4 and **(C)** T cell co-stimulation, **(D)** check-point, and **(E)** inflammation promoting. The linear regression to show the correlation between NFATC4 and **(F)** Tregs, **(G)** CD8^+^ T cells, and **(H)** plasma cells. Tregs, Regulatory T cells.

Immune genes are involved in immune responses via affecting immune cells; thus, we identified AML prognosis-related immune gene sets and immune cells by ssGSEA and CIBERSORT algorithm. As shown in Figures 6A,B, the correlation relationship between NFATC4 and AML prognosis-related immune gene sets and immune cells was presented by the heatmap. Figures 6C–E show the top three immune gene sets correlated with NFATC4. Among them, the correlation relationship between immune gene sets of T cell co-stimulation and NFATC4 was the most significant (*R* = 0.323, *P* < 0.001, positive), suggesting that NFATC4 might affect T cell co-stimulation in AML. The top three immune cells correlated with NFATC4 were Tregs (*R* = 0.526, *P* < 0.001, positive), CD8^+^ T cells (*R* = 0.339, *P* < 0.001, positive), and plasma cells (*R* = 0.263, *P* < 0.01, positive) (Figures 6F–H). Of them, the correlation relationship between NFATC4 and Tregs was most significant, as shown in Figure 6F, indicating that NFATC4 might modulate the cellular communication between leukemia cells and Tregs in the progression of AML.

### Multidimensional Validation Further Confirmed Association Between Key Biomarkers in Our Analysis With AML Prognosis

Multidimensional validation based on GEPIA (Supplementary Figure 3), Oncomine (Supplementary Figure 4), PROGgeneV2 (Supplementary Figure 5), UALCAN (Supplementary Figure 6), Linkedomics (Supplementary Figure 7), cBioportal (Supplementary Figure 8), GTEx (Supplementary Figure 9), UCSC xena (Supplementary Figure 10), CCLE (Supplementary Figure 11), Expression atlas, The Human Protein Altas (Supplementary Figure 12), and String (Supplementary Figure 13) was utilized.

The top five genes that represented the critical KEGG pathway were NSR, INS, PDX1, RBFOX2, and HNF1A. The genes' interaction relationship from the cBioportal database is shown in Supplementary Table 1. The differential expression of genes is summarized in Supplementary Table 2. RAG1 (Supplementary Figures 4B, 8B, 10A), NFATC4 (Supplementary Figure 8A), RBFOX2 (Supplementary Figure 8) and HNF1A (Supplementary Figures 8E, 10A), and INSR (Supplementary Figures 3C, 4C, 6C, 8C, 10A, 11B) were highly expressed in AML. PDX1 (Supplementary Figures 4C, 10A, 11C) was lowly expressed in AML. The validation of association between these genes and prognosis is summarized in Supplementary Table 3. The integrated genes (*P* < 0.05, PROGgeneV2, Supplementary Figure 5K), ISNR (*P* < 0.05, PROGgeneV2, Supplementary Figure 5J; *P* < 0.001, Linkedomics, Supplementary Figure 7C), and RBFOX2 (*P* < 0.05, GEPIA, Supplementary Figure 3) were significantly related to overall survival, and INSR (*P* < 0.05, cBioportal, Supplementary Figure 8C) was also significantly related to disease/progression-free survival.

To better show our findings, we constructed a schematic diagram of this scientific hypothesis (Supplementary Figure 14C). The crucial TF, immune gene, downstream pathway, and associated immune gene set and immune cells were RAG1, NFATC4, ABC transporter signaling pathway and T cell co-stimulation and Tregs, respectively.

## Discussion

Immune imbalance plays important roles in the progression of AML. However, the crosstalk between leukemia cells and immune cells, the critical participant of immune responses, remains elusive. Previous studies of immune responses have a limited view to a specific subset of immune cells to explore how they were regulated by leukemia cells. This may be misleading and are not comprehensive as various immune cells surrounding cancer cells are important. In the current study, we focused on expression of immune genes in leukemia cells and applied the CIBERSORT tool to explore the communication between leukemia cells and immune cells. Finally, NFATC4 was the key immune gene in poor prognosis of AML through recruiting Tregs.

In this study, RAG1 was found to be positively correlated with NFATC4 in the process of searching for key immune genes through co-expression analysis. Thus, we concluded that RAG1 transcriptionally regulated the expression of NFATC4. RAG1 is a key component of the RAG complex which is the main driving factor of oncogenic genome deletion and translocation (Han et al., 2019). High expression of RAG1 was associated with high proliferation markers in adult ALL and poor prognosis in gastric cancer (Han et al., 2019; Kang et al., 2019), which revealed the role of RAG1 in cancer progression. The transcriptional function of RAG1 for NFATC4 has not been described previously. However, the list of cancer-related TFs in our analysis was from Cistrome Cancer, a comprehensive resource for predicted TF targets in cancer. The prediction was based on TCGA expression profiles and public Chip-seq profiles. Therefore, we speculated that RAG1 was a transcription factor of NFATC4 in AML, while its transcriptional regulatory function needs further experimental verification.

The NFAT proteins were widely concerned in the immune system, while recent studies indicated that they are functionally active in several nonimmune cells and participate in tumor progression (Baksh et al., 2002; Graef et al., 2003; Neal and Clipstone, 2003). Our study discovered that high expression of NFATC4 was associated with poor prognosis of AML, which was consistent with reports in pancreatic cancer and ovarian cancer (Hessmann et al., 2016; Cole et al., 2020). In these tumors, NFATC4 participates in cancer progression through promoting tumor cell proliferation or chemotherapy resistance, while we inferred that it regulates immune responses in the progression of AML. Another isotype of NFATs, NFAT1, increases neutrophil infiltration through promoting the transcriptional induction of IL8 in breast cancer (Kaunisto et al., 2015). This indicates the role of NFAT family to regulate immune cells in cancer development. Besides, NFATC4 is reported to induce TNF-α expression in lung cells apart from involving in transcription of TNF-α in immune cells (Ke et al., 2006; Falvo et al., 2008). The repressed NFATC4 transcription activity in adipocytes also inhibited the secretion of inflammatory factors (Kim et al., 2010). Moreover, it is worth noting that NFATC4 signaling mediates the expression of chemokines CCL2 and CXCL10 in rat fibroblasts (Kuwata et al., 2018). Also, CCL2 was reported to recruit Tregs in the progression of esophageal squamous cell carcinoma (Yue et al., 2020). In this study, we found that Tregs were positively associated with NFATC4 in AML by CIBERSORT and Pearson correlation analysis, indicating that NFATC4 might involve in the progression of AML through recruiting Tregs.

As a nuclear factor, NFATC4 needs to activate downstream pathways to mediate the crosstalk between leukemia cells and Tregs. Pearson correlation analysis showed that NFATC4 was positively associated with ABC transporters, identified by GSVA and GSEA. In a previous study, NFATC2 promoted the downregulation of ABCA1 in an innate immunity signaling process, proving that NFATs could regulate ABC transporters (Maitra et al., 2009). Thus, we inferred that NFATC4 could enhance the expression of ABC transporters in AML. ABC transporters represent one of the largest transmembrane protein families, consisting of seven gene subfamilies (Begicevic and Falasca, 2017). Some ABC transporters participate in metabolite transportation, drug efflux, antigen processing, and immunity (Fukuda et al., 2015; Liu, 2019). So they may mediate the excretion of inflammatory factors to assist leukemia cells in recruiting Tregs. Furthermore, we found that the immune gene set of T cell co-stimulation was positively associated with NFATC4 in AML by ssGSEA and Pearson correlation analysis. One member in this immune gene set, TNFSF14, was known as a costimulatory factor for the activation of lymphoid cells and stimulation of the proliferation of T cells. The expression of TNFSF14 in melanoma cells contributes to regulate T-cell responses to tumor cells (Mortarini et al., 2005). Thus, we speculated that NFATC4 might affect the activation of Tregs through modulating the immune gene set of T cell co-stimulation.

To be honest, there are some limitations in our study. Firstly, the expression profiles and clinical information of samples in public database are limited. Besides, all the data for our speculation was from public databases, which lacked validation experiments. However, our study is a correlation analysis, aiming to provide reliable guidance for fundamental research of AML. Moreover, we also performed multidimensional online validation to support our results. All in all, our study indicated that increased NFATC4 might recruit Tregs in the progression of AML through affecting ABC transporters and T cell co-stimulation (Supplementary Figure 13). Further experiments will be carried out to verify our hypothesis.

## Conclusions

Our study, firstly, inferred that NFATC4 was key immune gene associated with poor prognosis of AML through recruiting Tregs. Our findings further uncover the mechanism of AML progression and might provide guidance for its treatment.

## Data Availability Statement

The original contributions presented in the study are included in the article/Supplementary Materials, further inquiries can be directed to the corresponding author/s.

## Author Contributions

CZ, SY, WL, JL, YW, HG, YZ, and JS: conception/design and final approval of manuscript. CZ and SY: collection and/or assembly of data. CZ: data analysis and interpretation. CZ, SY, and JS: manuscript writing. All authors contributed to the article and approved the submitted version.

## Conflict of Interest

The authors declare that the research was conducted in the absence of any commercial or financial relationships that could be construed as a potential conflict of interest.

## Abbreviations

AML: Acute myeloid leukemia
ABC transporters: ATP binding cassette transporters
AUC: Area Under the Curve
DEGs: Differentially expressed genes
GSEA: Gene set enrichment analysis
GSVA: Gene set variation analysis
GO: Go Ontology
KEGG: Kyoto Encyclopedia of Genes and Genomes
KIR2DL4: Killer cell immunoglobulin-like receptor 2DL4
KLC3: Kinesin light chain 3
NFATC4: Nuclear factor activated T cells-4
NCR2: Natural cytotoxicity triggering receptor 2
NPDC1: Neural proliferation, differentiation and control 1
RAG1: Recombination activating gene-1
Tregs: Regulatory T cells
ssGSEA: Single sample gene set enrichment analysis.
