# Supplementary material for: Increased NFATC4 Correlates With Poor Prognosis of AML Through Recruiting Regulatory T Cells
Source: Front Genet. 2020 Nov 27;11:573124. doi: 10.3389/fgene.2020.573124 (PMC7728998; doi:10.3389/fgene.2020.573124)
Supplement: Supplementary file 1 [file Data_Sheet_1.docx]

# Supplementary Material

**Supplementary Table 1** Mutual Exclusivity of NFATC4, RAG1, INSR, INS, PDX1, RBFOX2 and HNF1A in cBioportal database

| A | B | Neither | A Not B | B Not A | Both | Log2 Odds Ratio | p-Value | q-Value | Tendency |
| --- | --- | --- | --- | --- | --- | --- | --- | --- | --- |
| RAG1 | INS | 1281 | 4 | 1 | 1 | >3 | 0.008 | 0.163 | Co-occurrence |
| NFATC4 | INSR | 1277 | 2 | 7 | 1 | >3 | 0.019 | 0.195 | Co-occurrence |
| INSR | PDX1 | 1274 | 8 | 5 | 0 | <-3 | 0.969 | 1 | Mutual exclusivity |
| RAG1 | INSR | 1274 | 5 | 8 | 0 | <-3 | 0.969 | 1 | Mutual exclusivity |
| INSR | RBFOX2 | 1275 | 8 | 4 | 0 | <-3 | 0.975 | 1 | Mutual exclusivity |
| RAG1 | PDX1 | 1277 | 5 | 5 | 0 | <-3 | 0.981 | 1 | Mutual exclusivity |
| RAG1 | RBFOX2 | 1278 | 5 | 4 | 0 | <-3 | 0.985 | 1 | Mutual exclusivity |
| PDX1 | RBFOX2 | 1278 | 5 | 4 | 0 | <-3 | 0.985 | 1 | Mutual exclusivity |
| INSR | INS | 1277 | 8 | 2 | 0 | <-3 | 0.988 | 1 | Mutual exclusivity |
| NFATC4 | RAG1 | 1279 | 3 | 5 | 0 | <-3 | 0.988 | 1 | Mutual exclusivity |
| NFATC4 | PDX1 | 1279 | 3 | 5 | 0 | <-3 | 0.988 | 1 | Mutual exclusivity |
| NFATC4 | RBFOX2 | 1280 | 3 | 4 | 0 | <-3 | 0.991 | 1 | Mutual exclusivity |
| INS | PDX1 | 1280 | 2 | 5 | 0 | <-3 | 0.992 | 1 | Mutual exclusivity |
| INS | RBFOX2 | 1281 | 2 | 4 | 0 | <-3 | 0.994 | 1 | Mutual exclusivity |
| NFATC4 | INS | 1282 | 3 | 2 | 0 | <-3 | 0.995 | 1 | Mutual exclusivity |
| NFATC4 | HNF1A | 1284 | 3 | 0 | 0 | >3 | 1 | 1 | Co-occurrence |
| RAG1 | HNF1A | 1282 | 5 | 0 | 0 | >3 | 1 | 1 | Co-occurrence |
| INSR | HNF1A | 1279 | 8 | 0 | 0 | >3 | 1 | 1 | Co-occurrence |
| INS | HNF1A | 1285 | 2 | 0 | 0 | >3 | 1 | 1 | Co-occurrence |
| PDX1 | HNF1A | 1282 | 5 | 0 | 0 | >3 | 1 | 1 | Co-occurrence |
| RBFOX2 | HNF1A | 1283 | 4 | 0 | 0 | >3 | 1 | 1 | Co-occurrence |

**Supplementary Table 2** Summary of multidimensional external validation results of gene expression based on multiple databases

|  | **NFATC4** | | **RAG1** | | **INSR** | | **INS** | | **PDX1** | | **RBFOX2** | | **HNF1A** | | **Results** |
| --- | --- | --- | --- | --- | --- | --- | --- | --- | --- | --- | --- | --- | --- | --- | --- |
|  | **N** | **L** | **N** | **L** | **N** | **L** | **N** | **L** | **N** | **L** | **N** | **L** | **N** | **L** |  |
| **GEPIA** | **-** | **↓** | **-** | **↓** | **-** | **↑** | **-** | **ND** | **-** | **-** | **↑** | **-** | **-** | **-** | NFAT4 and RAG1 low-expressed, while INSR high-expressed in AML (figure S3). |
| **Oncomine** | **NA** | **↓** | **NA** | **↑** | **NA** | **↑** | **NA** | **↓** | **NA** | **↓** | **NA** | **↓** | **NA** | **↓** | NFATC4, INS, PDX1, RBFOX2 and HNF1A low-expressed, while RAG1 and INSR high- expressed in AML (figure S4). |
| **PROGgeneV2** | **NA** | **-** | **NA** | **-** | **NA** | **↓** | **NA** | **-** | **NA** | **-** | **NA** | **-** | **NA** | **-** | INSR low-expressed in AML (figure S5). |
| **UALCAN** | **NA** | **↓** | **NA** | **-** | **NA** | **↑** | **NA** | **NA** | **NA** | **NA** | **NA** | **NA** | **NA** | **↓** | NFATC4 and HNF1A low-expressed, while INSR high-expressed in AML (figure S6). |
| **Linkedomics** | **NA** | **-** | **NA** | **-** | **NA** | **↓** | **NA** | **NA** | **NA** | **-** | **NA** | **NA** | **NA** | **--** | INSR low expressed in AML (figure S7). |
| **cBioportal** | **NA** | **↑** | **NA** | **↑** | **NA** | **↑** | **NA** | **↓** | **NA** | **-** | **NA** | **↑** | **NA** | **↑** | NFATC4, RAG1, INSR, RBFOX2 and HNF1A high-expressed, while INS low-expressed in AML (figure S8). |
| **GTEx** | **-** | **NA** | **-** | **NA** | **↑** | **NA** | **-** | **NA** | **-** | **NA** | **-** | **NA** | **-** | **NA** | INSR high expressed in normal bone marrow (figure S9). |
| **UCSC xena** | **NA** | **-** | **NA** | **↑** | **NA** | **↑** | **NA** | **↓** | **NA** | **↓** | **NA** | **NA** | **NA** | **↑** | RAG1, INSR and HNF1A high-expressed, while INS and PDX1 low-expressed in AML (figure S10). |
| **CCLE** | **NA** | **↓** | **NA** | **↓** | **NA** | **↑** | **NA** | **↓** | **NA** | **↓** | **NA** | **-** | **NA** | **↓** | NFATC4, RAG1, INS, PDX1 and HNF1A low-expressed, while INSR high-expressed in AML cell line (figure S11). |
| **Expression atlas** | **NA** | **NA** | **NA** | **NA** | **NA** | **NA** | **NA** | **NA** | **NA** | **NA** | **NA** | **NA** | **NA** | **NA** | none |
| **The human protein atlas** | **↓** | **↓** | **NA** | **NA** | **ND** | **↓** | **ND** | **↓** | **ND** | **ND** | **-** | **↑** | **ND** | **NA** | NFATC4 low-expressed in normal bone marrow and tumor tissue, INSR and INS low-expressed in tumor tissue, RBFOX2 high-expressed in tumor tissue (figure S12). |

**Note:** “N” was defined as normal; “L” was defined as Acute Myeloid Leukemia;“↑” was defined as a

significantly high-expressed gene; “↓” was defined as a significantly low-expressed gene; “NA” was defined as “Not available”; “ND” was defined as “Not detached”; “-” was defined as a gene with no significant difference in expression.

**Abbreviations:** AML, Acute Myeloid Leukemia; GTEx, Genotype-Tissue Expression; CCLE, Cancer Cell Line Encyclopedia; GEPIA, Gene Expression Profilling Interactive Analysis.

**Supplementary Table 3** Summary of multidimensional external validation results of prognosis based on multiple databases

|  | **NFATC4** | **RAG1** | **INSR** | **INS** | **PDX1** | **RBFOX2** | **HNF1A** | **Results** |
| --- | --- | --- | --- | --- | --- | --- | --- | --- |
| **GEPIA** | OS  P = 0.210 | OS  P = 0.075 | OS  P = 0.130 | NA | NA | OS  P = 0.022 | OS  P = 0.690 | RBFOX2 was significant related to overall survival (figure S3). |
| **PROGgeneV2** | GSE12417 U133B: OS P = 0.180  TCGA: OS  P = 0.899 | GSE12417 U133B: NA  TCGA: OS  P = 0.115 | GSE12417 U133B: OS P = 0.102  TCGA: OS  P = 0.007 | GSE12417 U133B: NA  TCGA: OS  P = 0.199 | GSE12417 U133B: NA  TCGA: OS  P = 0.092 | GSE12417 U133B: NA  TCGA: OS  P = 0.292 | GSE12417 U133B: NA  TCGA: OS  P = 0.365 | INSR was significant related to overall survival, and integrated genes also related to overall survival (figure S5). |
|  | GSE12417 U133B: The integrated genes OS P = 0.025  TCGA: The integrated genes OS P = 0.225 | | | | | | |  |
| **Linkedomics** | OS P = 0.796 | OS P = 0.152 | OS P＜ 0.001 | NA | OS P = 0.959 | NA | OS P = 0.615 | INSR was significant related to overall survival (figure S7). |
| **cBioportal** | OS  P = 0.342  NA | OS P = 0.581  D/PF  P = 0.270 | OS P = 0.918  D/PF  P = 0.027 | OS P = 0.397 NA | OS P = 0.082  D/PF  P = 0.136 | OS P = 0.063  D/PF  P = 0.214 | OS P = 0.347  NA | INSR was significant related to prognosis (figure S8). |
|  | The integrated genes OS P = 0.608, D/PF P = 0.523 | | | | | | |  |

**Abbreviations:** OS, Overall survival; D/PF, Disease/Progression-free; NA, Not available.

Supplementary Figure 1 The flowchart of study process.

Supplementary Figure 2 The non-parametric tests among favorable, intermediate and poor prognosis AML. *P < 0.05.

Supplementary Figure 3 The expression level of NFATC4 (A), RAG1 (B), INSR (C), PDX1 (D), RBFOX2 (E) and HNF1A (F) in AML. The overall survival K-M curves of NFATC4 (G), RAG1 (H), INSR (I), RBFOX2 (J) and HNF1A (K).

Supplementary Figure 4 The expression level of NFATC4 (A), RAG1 (B), INSR (C), INS (D), PDX1 (E), RBFOX2 (F) and HNF1A (G) in AML.

Supplementary Figure 5 The overall survival K-M curves of NFATC4 (A), RAG1 (B), INSR (C), INS (D), PDX1 (E), RBFOX2 (F), HNF1A (G) and integrated genes (H) in TCGA in AML. The overall survival K-M curves of NFATC4 (I), INSR (J) and integrated genes (K) in GSE12417-U133B in AML.

Supplementary Figure 6 The expression level of NFATC4 (A), RAG1 (B), INSR (C) and HNF1A (D) in AML. The overall survival K-M curves of NFATC4 (E), RAG1 (F), INSR (G) and HNF1A (H) in AML.

Supplementary Figure 7 The overall survival K-M curves of NFATC4 (A), RAG1 (B), INSR (C), PDX1 (D) and HNF1A (E) in AML. (F-G) The correlation relationship between NFATC4 and proteins in RPPA.

Supplementary Figure 8 The overall survival K-M curves of NFATC4 (A), RAG1 (B), INSR (C), INS (D) and PDX1 (E), RBFOX2 (F), HNF1A (G) and integrated genes (H) in AML. The disease/progression free survival K-M curves of RAG1 (I), INSR (J), PDX1 (K), RBFOX2 (L), and integrated genes (M) in AML.

Supplementary Figure 9 The heatmap of expression level for NFATC4 (A), RAG1 (B), INSR (C), INS (D), PDX1 (E), RBFOX2 (F) and HNF1A (G) in normal tissue.

Supplementary Figure 10 The expression level (A), the PCA plot (B) and the heatmap (C) of NFATC4, RAG1, INSR, INS, PDX1, RBFOX2 and HNF1A in AML.

Supplementary Figure 11 The expression level of NFATC4 (A), RAG1 (B), INSR (C), INS (D), PDX1 (E), RBFOX2 (F) and HNF1A (G) in AML cell line.

Supplementary Figure 12 The expression level of NFATC4 (A), INSR (B), INS (C), PDX1 (D), RBFOX2 (E) and HNF1A (F) in normal bone marrow and tumor tissue.

Supplementary Figure 13 The protein-protein interaction network of NFATC4, RAG1, INSR, INS, PDX1, RBFOX2 and HNF1A.

Supplementary Figure 14 The network of this scientific hypothesis including key TF, immune gene, downstream immune gene set and pathway. (A) The protein-protein interaction network of key TF, immune gene, downstream KEGG pathways, immune gene sets and immune cells. (B) Co-expressed circle plot of KEGG pathways. (C) Schematic diagram of the scientific hypothesis about NFATC4 Regulated by RAG1 affects ABC transporter pathway, immune gene set of check point and Tregs in the poor prognosis of AML based on analysis in silico.
