# Supplementary figures and images for: Increased NFATC4 Correlates With Poor Prognosis of AML Through Recruiting Regulatory T Cells
Source: Front Genet. 2020 Nov 27;11:573124. doi: 10.3389/fgene.2020.573124 (PMC7728998; doi:10.3389/fgene.2020.573124)

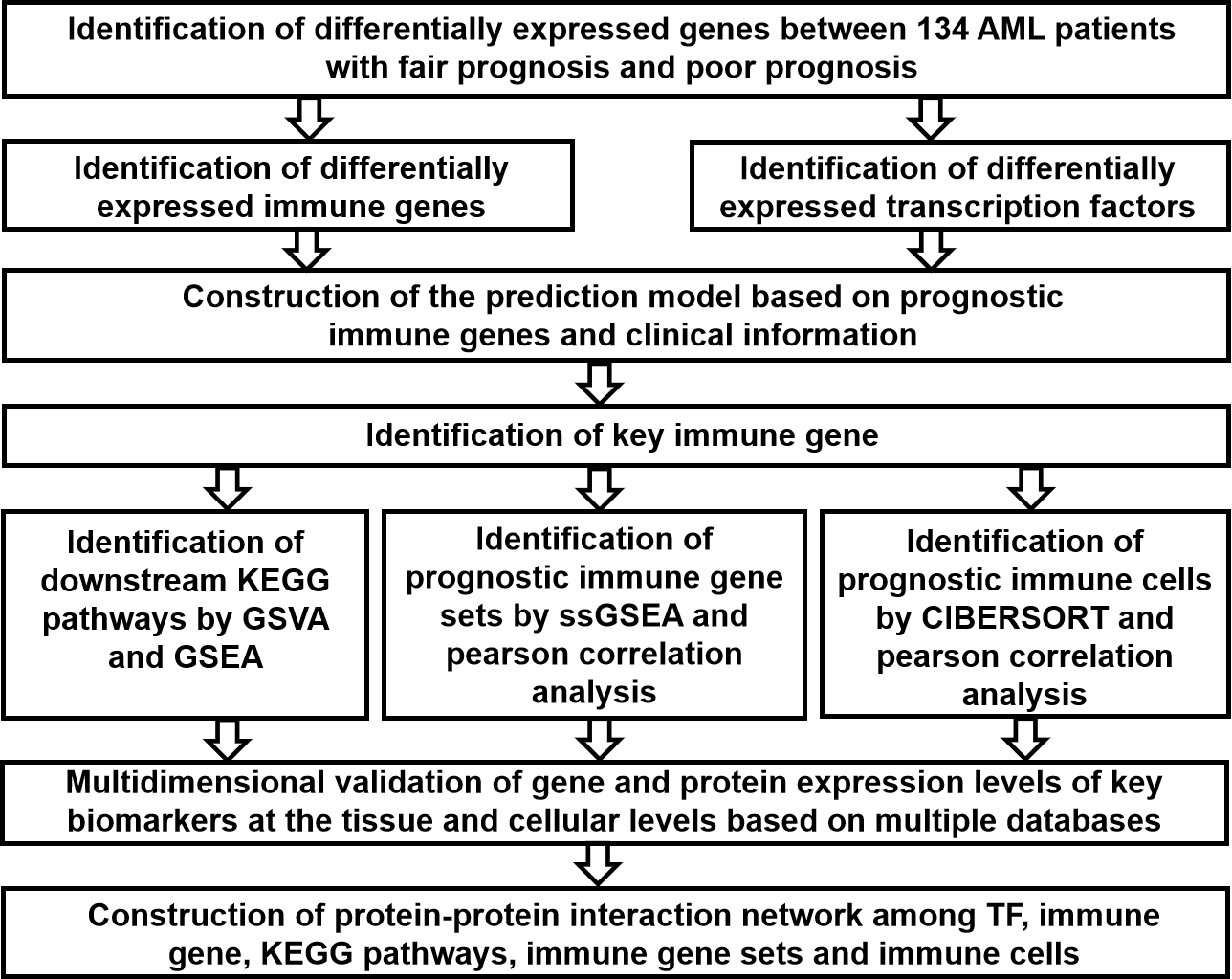

Supplement: Supplementary file 2 [file Image_1.TIF]

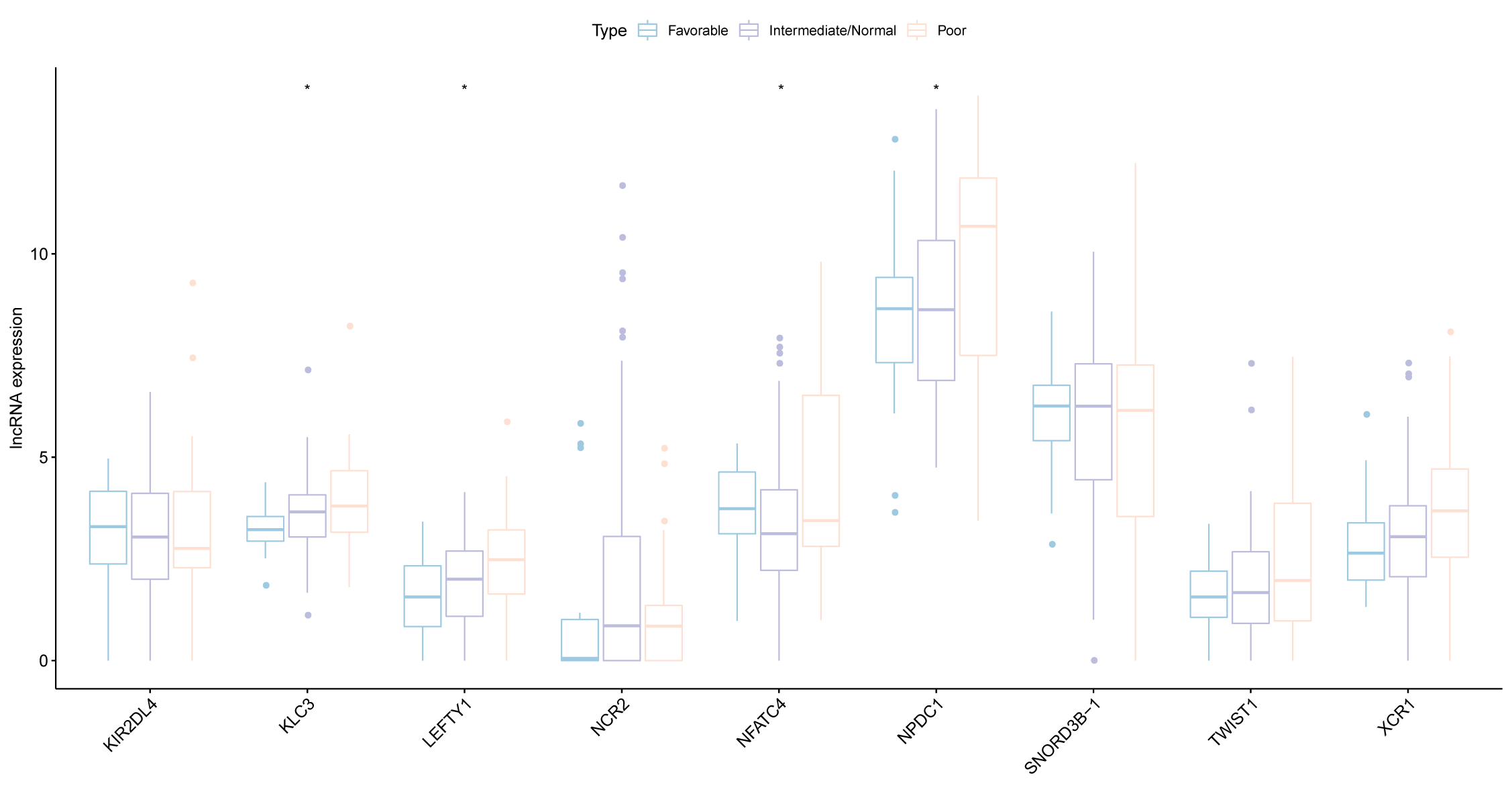

Supplement: Supplementary file 3 [file Image_2.TIF]

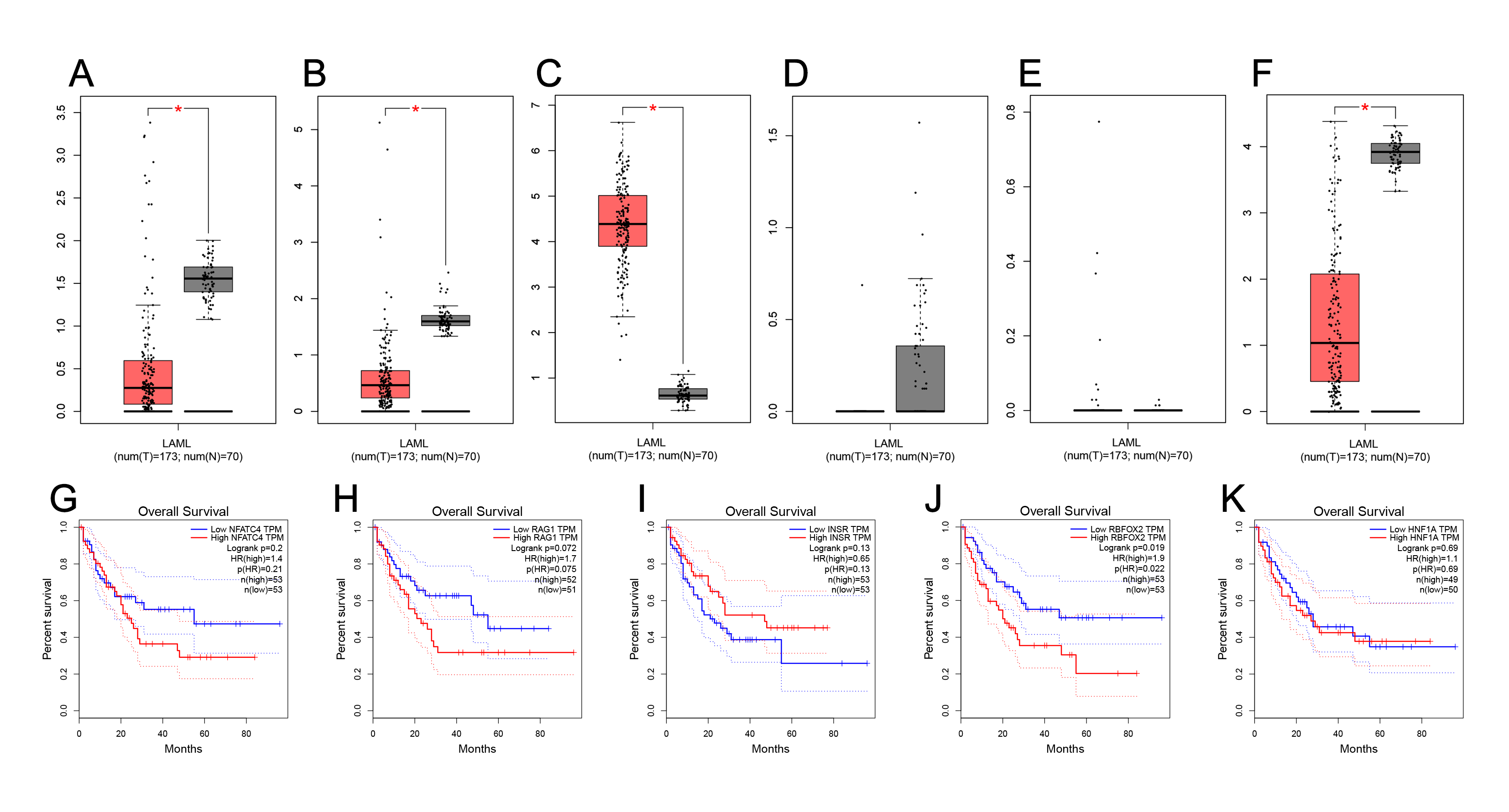

Supplement: Supplementary file 4 [file Image_3.TIF]

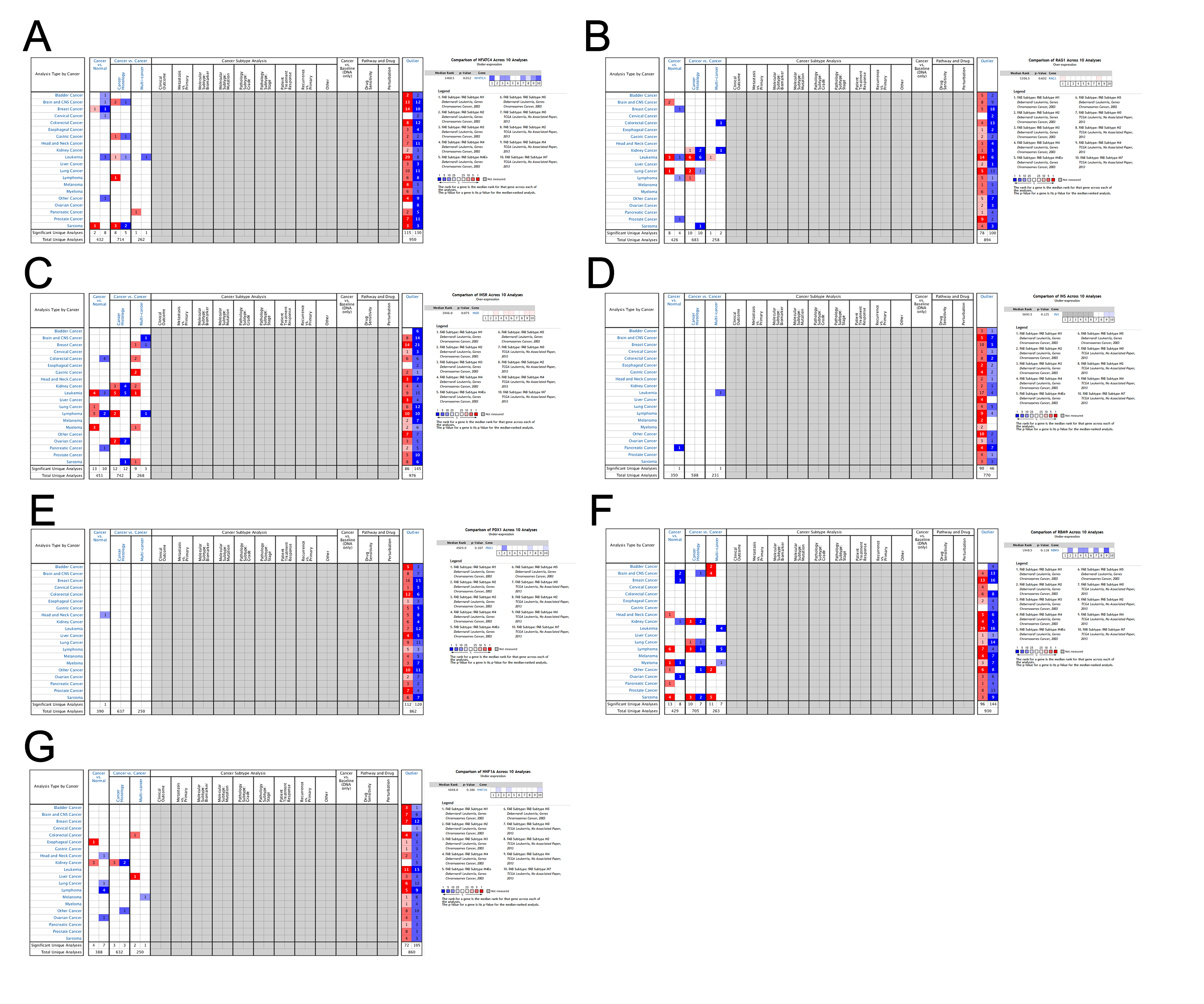

Supplement: Supplementary file 5 [file Image_4.TIF]

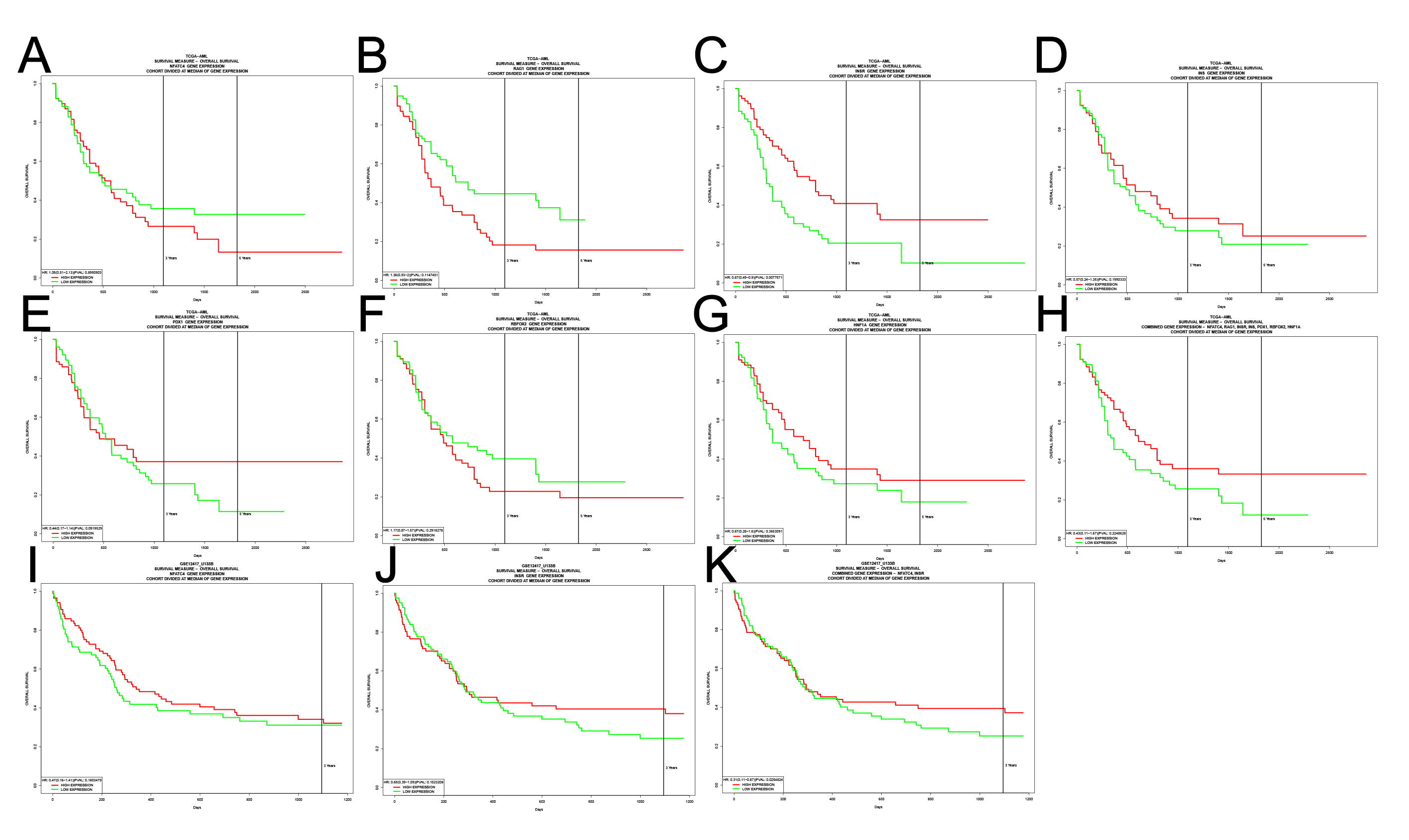

Supplement: Supplementary file 6 [file Image_5.TIF]

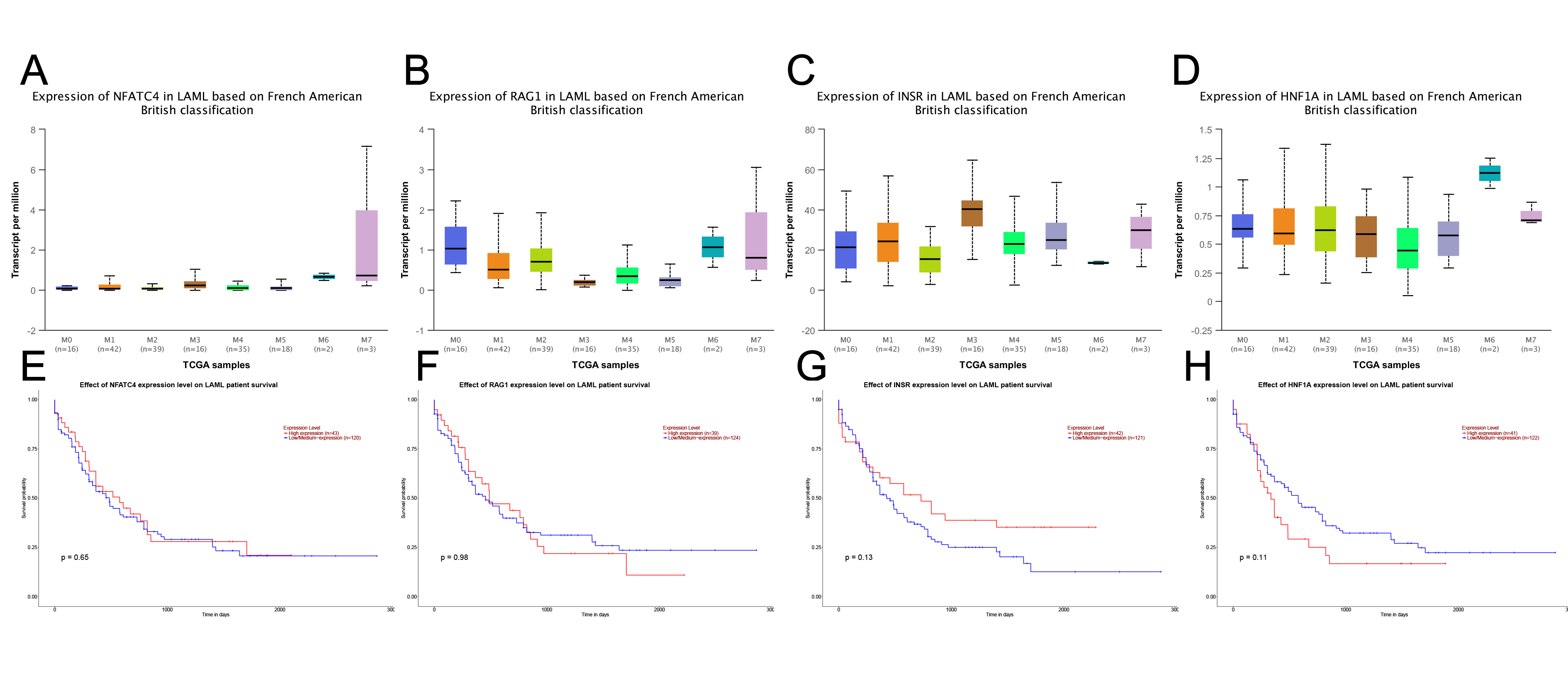

Supplement: Supplementary file 7 [file Image_6.TIF]

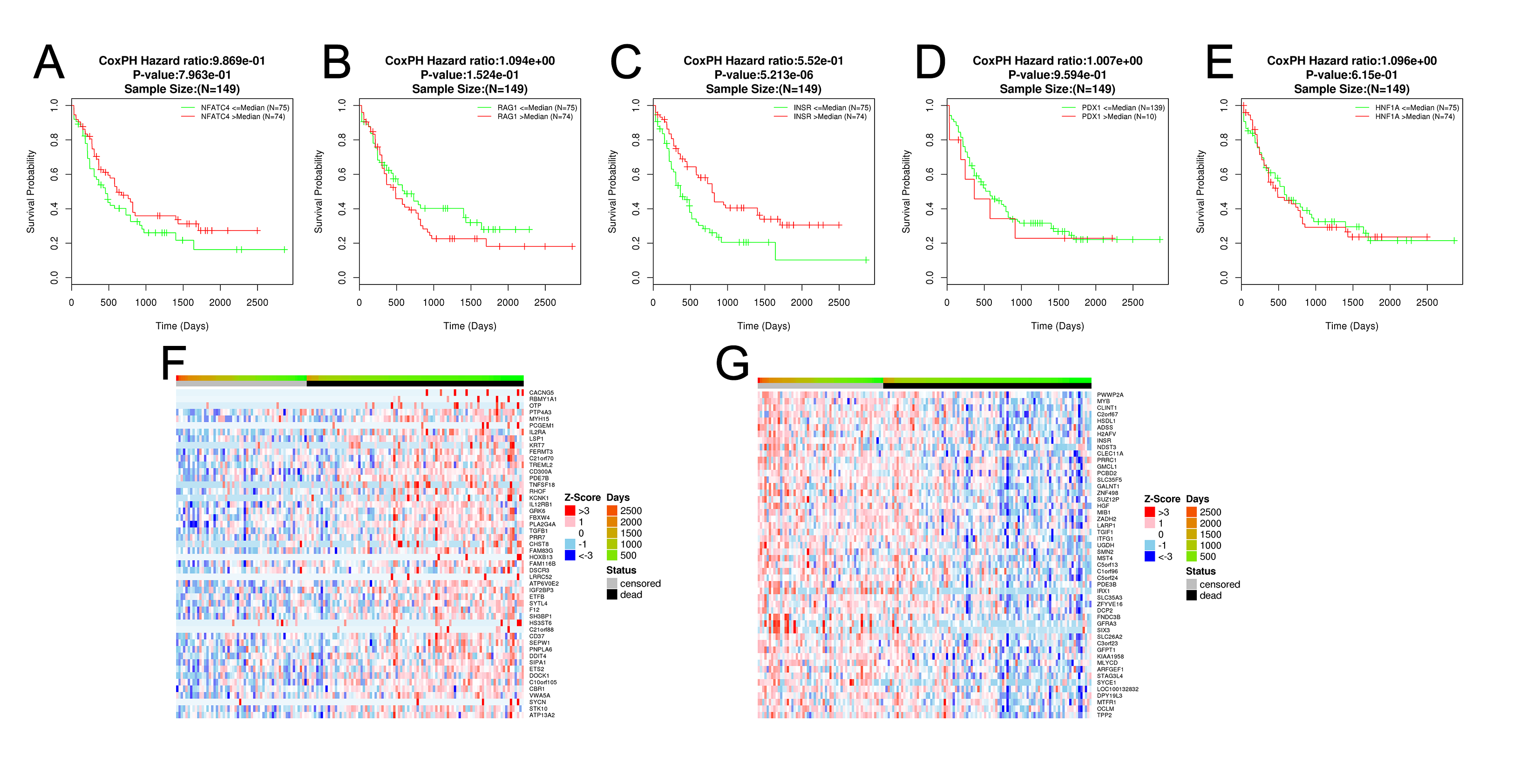

Supplement: Supplementary file 8 [file Image_7.TIF]

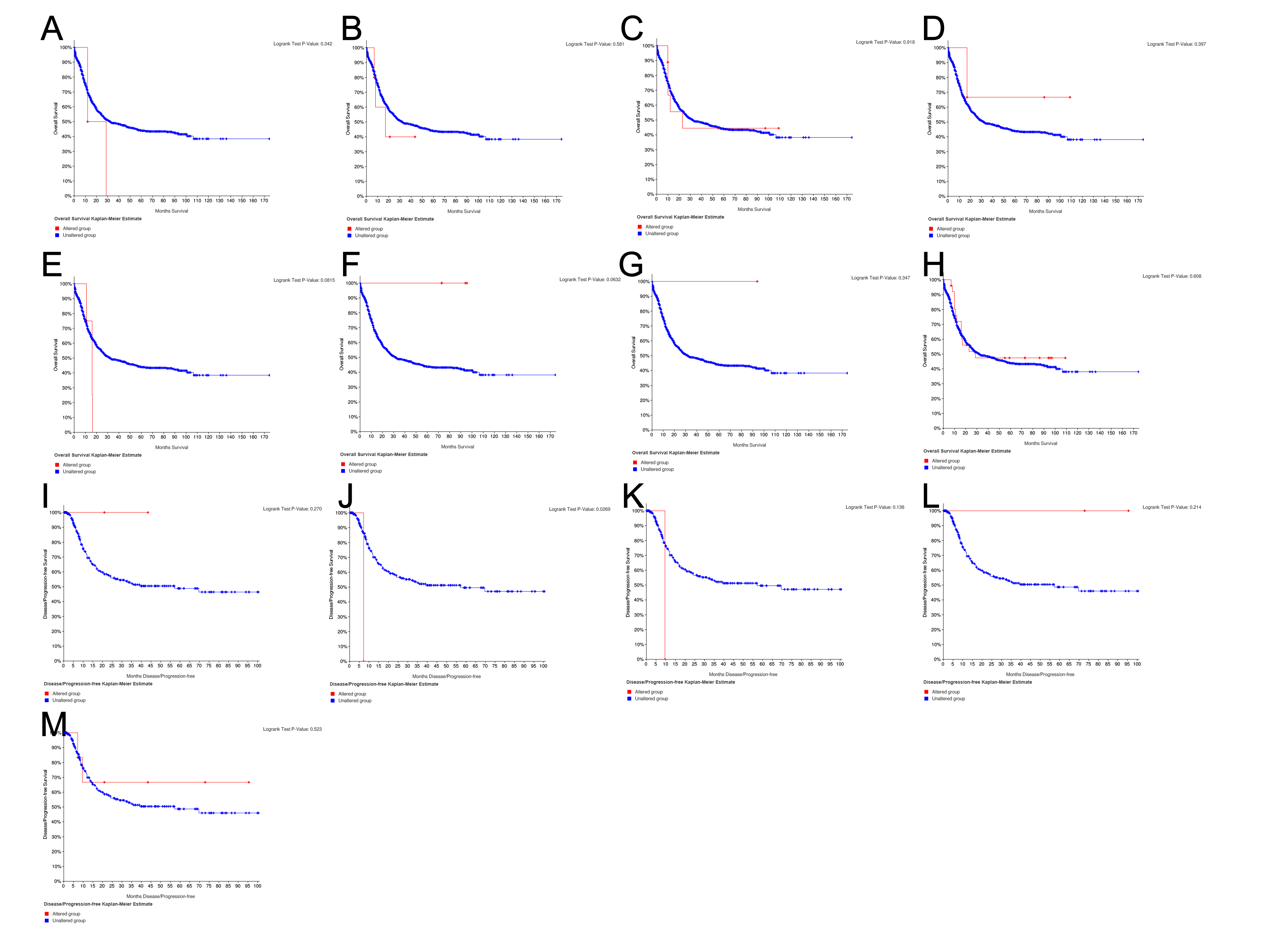

Supplement: Supplementary file 9 [file Image_8.TIF]

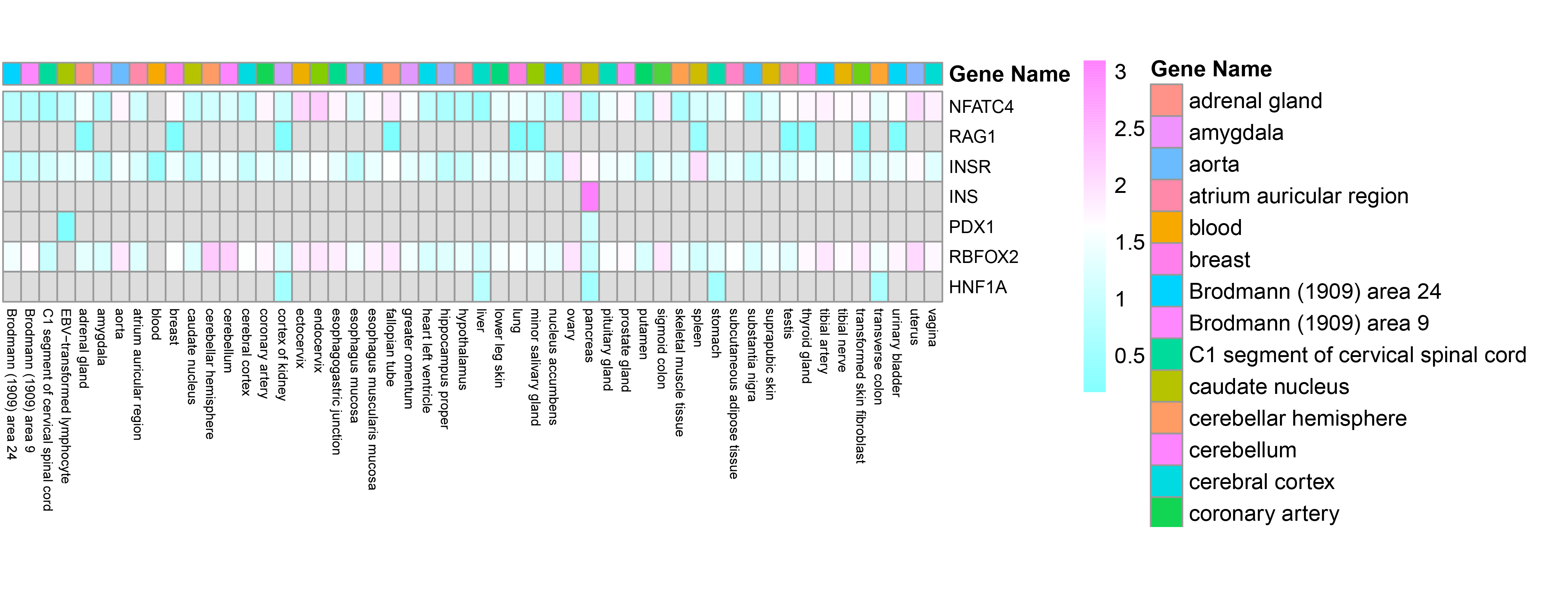

Supplement: Supplementary file 10 [file Image_9.TIF]

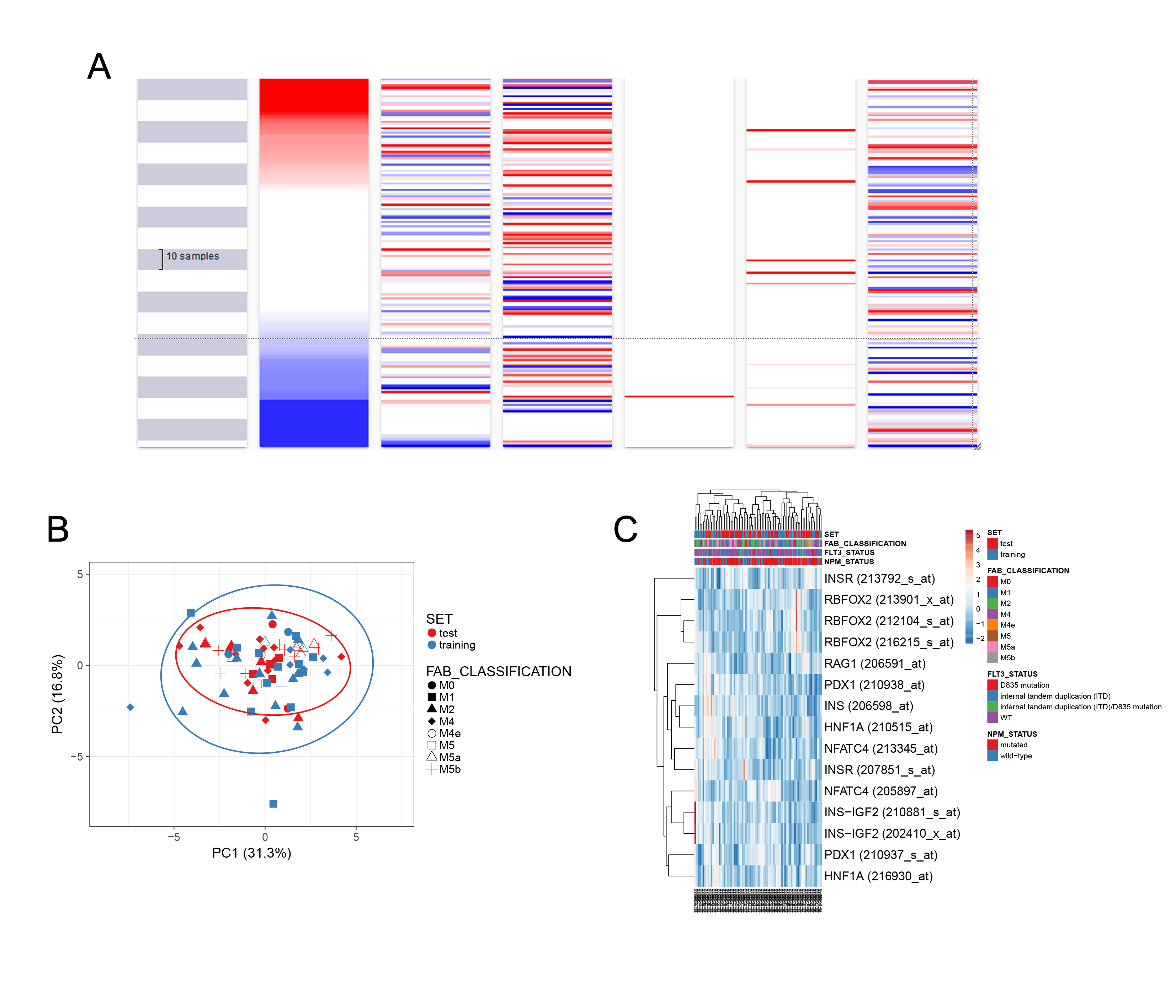

Supplement: Supplementary file 11 [file Image_10.TIF]

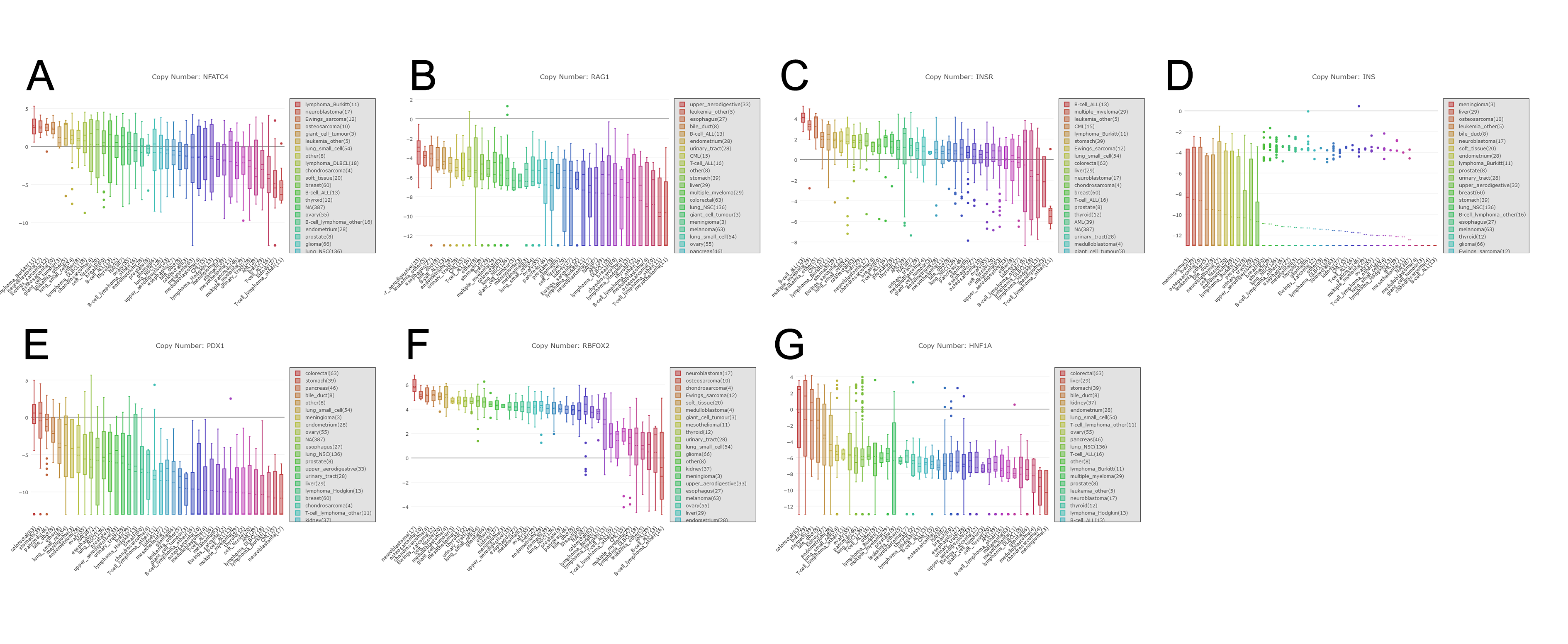

Supplement: Supplementary file 12 [file Image_11.TIF]

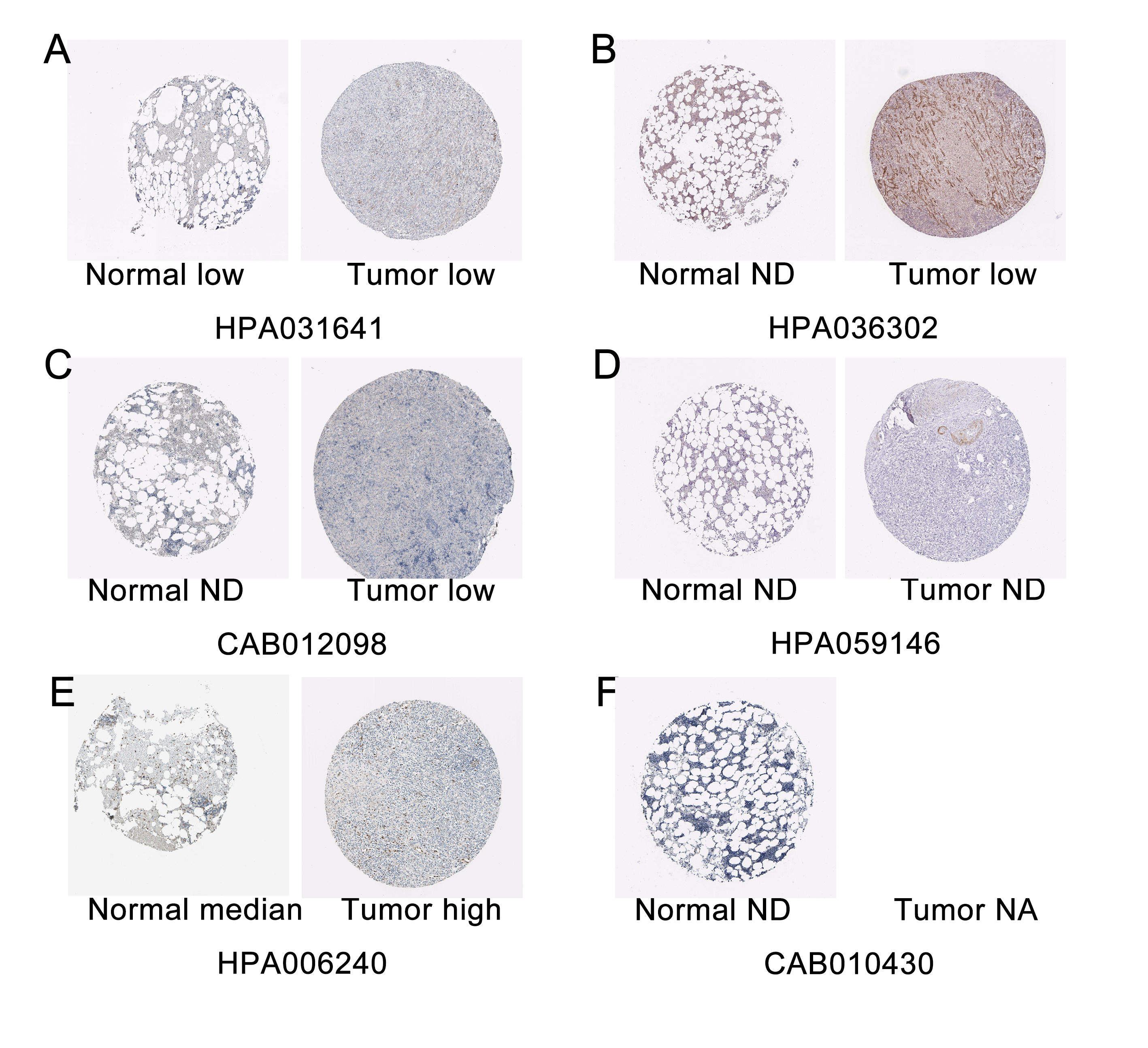

Supplement: Supplementary file 13 [file Image_12.TIF]

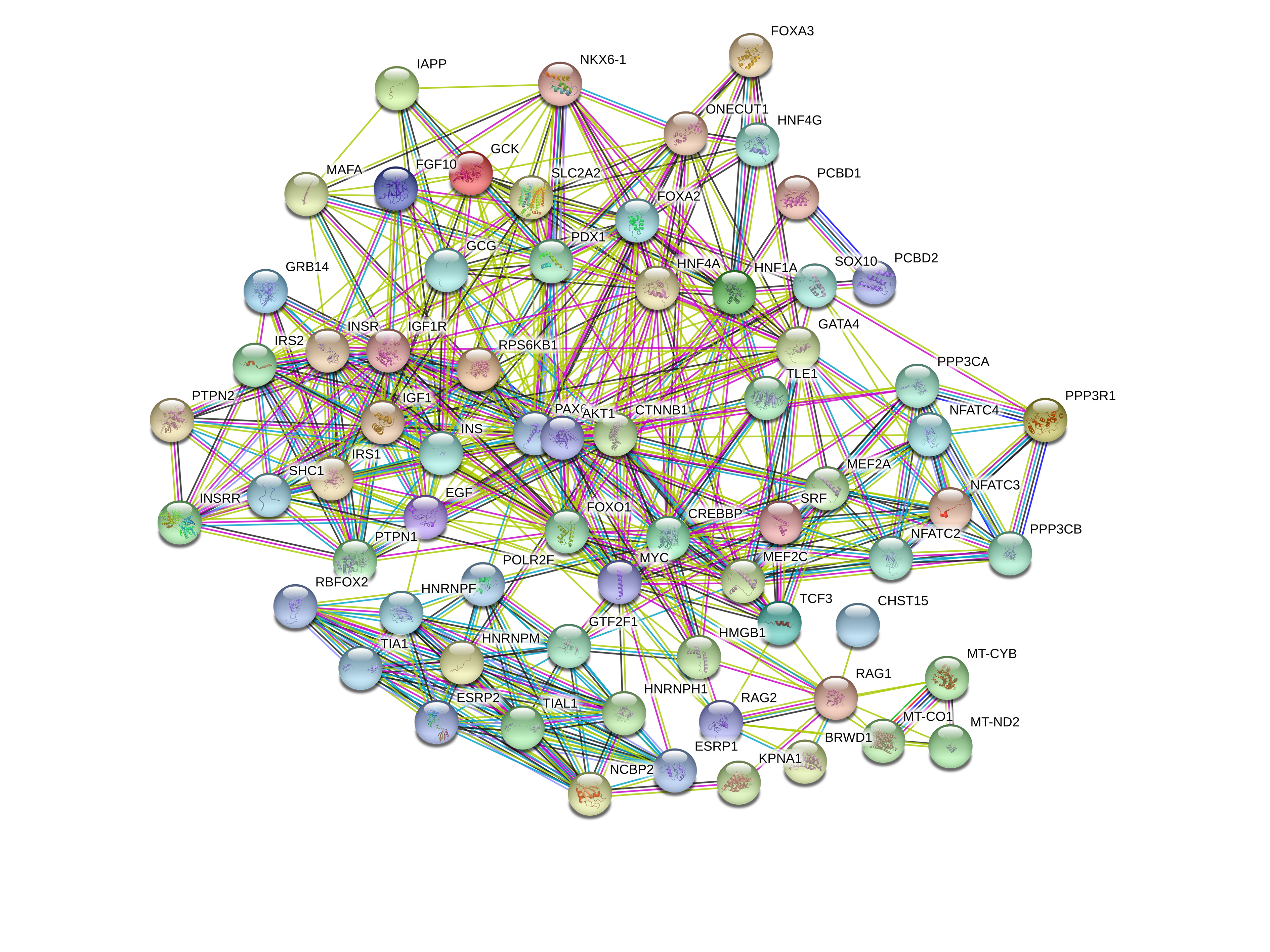

Supplement: Supplementary file 14 [file Image_13.TIF]

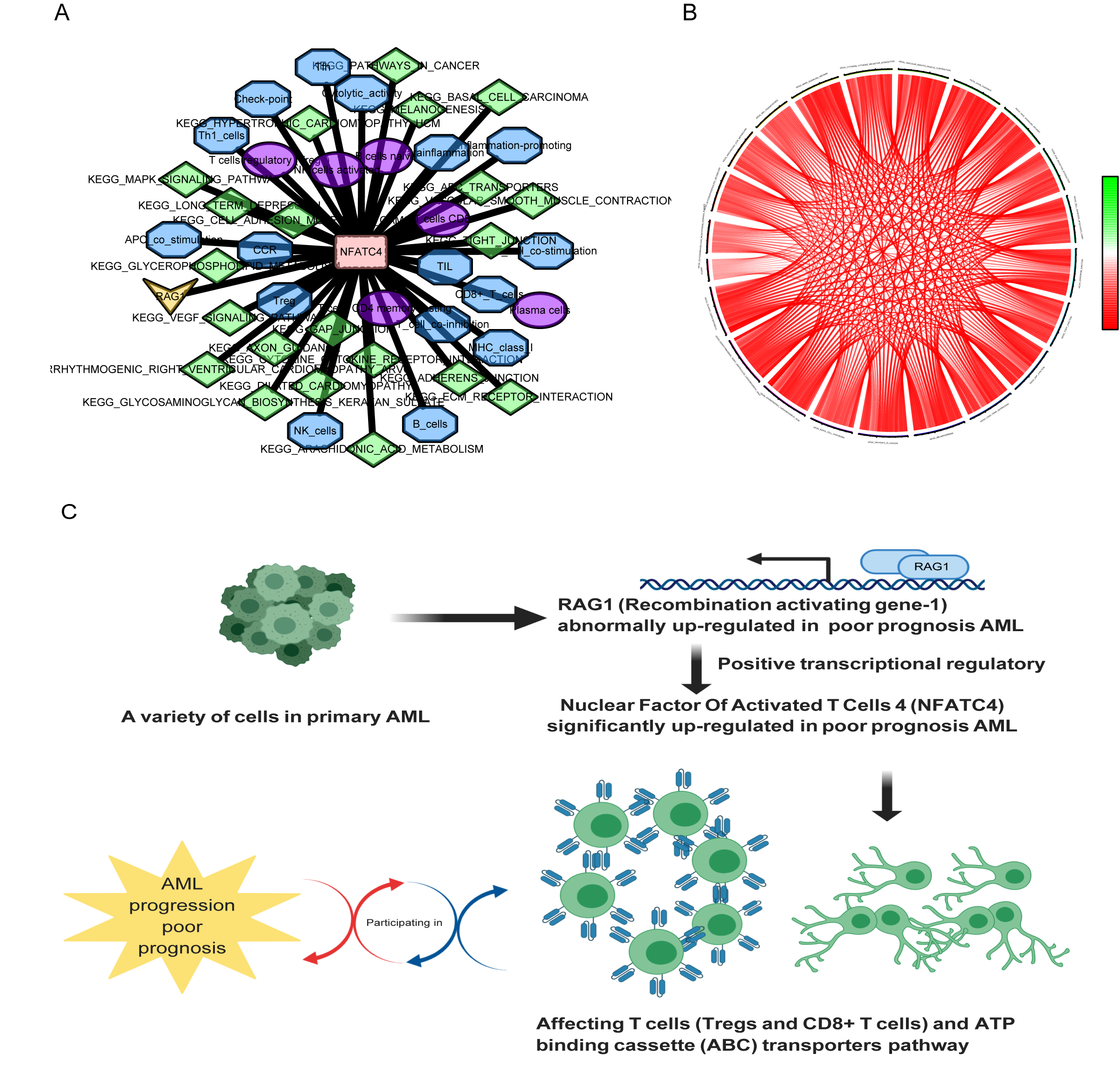

Supplement: Supplementary file 15 [file Image_14.TIF]
